# Can Online Consumers Contribute to Drug Knowledge? A Mixed-Methods Comparison of Consumer-Generated and Professionally Controlled Psychotropic Medication Information on the Internet

**DOI:** 10.2196/jmir.1716

**Published:** 2011-07-29

**Authors:** Shannon Hughes, David Cohen

**Affiliations:** ^1^Utah State UniversityDepartment of Sociology, Social Work, and AnthropologyLogan, UTUnited States; ^2^Florida International UniversityRobert Stempel College of Public Health and Social WorkMiami, FLUnited States

**Keywords:** Psychotropic drugs, mental health, consumer health information, Internet, pharmacoepidemiology, drug monitoring, product surveillance, postmarketing

## Abstract

**Background:**

Ongoing initiatives to filter online health searches exclude consumer-generated content from search returns, though its inferiority compared with professionally controlled content is not demonstrated. The antidepressant escitalopram and the antipsychotic quetiapine have ranked over the last 5 years as top-selling agents in their respective drug classes. Both drugs have various off-label mental health and non–mental health uses, ranging from the relief of insomnia and migraines to the treatment of severe developmental disorders.

**Objective:**

Our objective was to describe the most frequently reported effects of escitalopram and quetiapine in online consumer reviews, to compare them with effects described in professionally controlled commercial health websites, and to gauge the usability of online consumer medication reviews.

**Methods:**

A stratified simple random sample of 960 consumer reviews was selected from all 6998 consumer reviews of the two drugs in 2 consumer-generated (www.askapatient.com and www.crazymeds.us) and 2 professionally controlled (www.webmd.com and www.revolutionhealth.com) health websites. Professional medication descriptions included all standard information on the medications from the latter 2 websites. All textual data were inductively coded for medication effects, and intercoder agreement was assessed. Chi-square was used to test for associations between consumer-reported effects and website origination.

**Results:**

Consumers taking either escitalopram (n = 480) or quetiapine (n = 480) most frequently reported symptom improvement (30.4% or 146/480, 24.8% or 119/480) or symptom worsening (15.8% or 76/480, 10.2% or 49/480), changes in sleep (36% or 173/480, 60.6% or 291/480) and changes in weight and appetite (22.5% or 108/480, 30.8% or 148/480). More consumers posting reviews on consumer-generated rather than professionally controlled websites reported symptom worsening on quetiapine (17.3% or 38/220 versus 5% or 11/220, *P* < .001), while more consumers posting on professionally controlled websites reported symptom improvement (32.7% or 72/220 versus 21.4% or 47/220, *P* = .008). Professional descriptions more frequently listed physical adverse effects and warnings about suicidal ideation while consumer reviews emphasized effects disrupting daily routines and provided richer descriptions of effects in context. The most recent 20 consumer reviews on each drug from each website (n = 80) were comparable to the full sample of reviews in the frequency of commonly reported effects.

**Conclusion:**

Consumer reviews and professional medication descriptions generally reported similar effects of two psychotropic medications but differed in their descriptions and in frequency of reporting. Professional medication descriptions offer the advantage of a concise yet comprehensive listing of drug effects, while consumer reviews offer greater context and situational examples of how effects may manifest in various combinations and to varying degrees. The dispersion of consumer reviews across websites limits their integration, but a brief browsing strategy on the two target medications nonetheless retrieved representative consumer content. Current strategies for filtering online health searches to return only *trusted* or *approved* websites may inappropriately address the challenge to identify quality health sources on the Internet because such strategies unduly limit access to an entire complementary source for health information.

## Introduction

Consumers and clinicians increasingly consult consumer-generated health content on the Internet [1-3], but there are no direct comparisons of such content with that found on professionally controlled commercial health websites. Ongoing initiatives in Internet searching aim to filter health-related searches to return only sources meeting medical grading system requirements such as depth, timeliness, transparency, and readability [4,5]. These so-called trusted sources typically include broadly networked and well-resourced commercial, institutional, and government websites representing a professional knowledge base but exclude consumer-generated content [5-7]. Despite widespread discussion and speculation about the varying quality of health information on the Internet, such initiatives may be premature in the absence of reliable evidence suggesting that nonprofessionally delivered content is necessarily inferior to that provided by professionally controlled health sites [8,9].

In parallel, there is increasing momentum to gather patient-reported health and treatment outcomes [10,11], with the Internet identified as a major mechanism to accomplish this efficiently [12-14]. While much research has focused on developing and implementing new Internet-based technologies to collect patient-reported outcomes, studies on the practical uses of existing consumer-generated online health content remain limited in number and scope. In the mental health arena, for example, researchers have described discussion themes in online support groups [15], the efficacy of such groups to reduce depressive symptoms [16], online help-seeking behaviors [17], and completeness of drug information on pharmaceutical company websites [18-20]. An analysis of 1 year of comments from an online discussion forum identified 238 drug-related problems with antiparkinsonian agents, noting incongruences with clinical trial data [21]. Online consumer comments were also employed to analyze the subjective effects of older and newer antipsychotic medications [22]. Finally, the online patient community PatientsLikeMe collects longitudinal data on treatment effects for various conditions from their members and presents these data to the scientific community [23,24].

Although consumer-generated content about psychiatric medications may take many forms, much of it appears online as brief (usually 1 to 3 paragraphs) first-person accounts or reviews of experiences around the ingestion of a prescribed medication. This study describes, for two widely prescribed psychotropic drugs, the most frequently reported effects in online consumer reviews found both in consumer-generated and professionally controlled commercial health websites. It also compares consumer-reported effects of the two drugs to the authoritative account of these drugs’ effects found in professionally controlled commercial health websites. Moreover, it does so by privileging neither source as an a priori standard for quality and accuracy or by using standardized drug effect terminology. The findings provide the first empirical basis to evaluate possible advantages and disadvantages of using each online source (consumer-generated and professionally controlled health sites) for making medication-related treatment decisions.

## Methods

### Website Sampling

Since consumer-generated health content is unlikely to be returned among top search engine results, search engines and an index of online mental health resources compiled over the last 16 years by www.psychcentral.com were both employed to identify 2 consumer-generated websites. Combinations of the following search terms were used in Google and Yahoo search engines: patient, consumer, review, rating, support, and Lexapro (or Seroquel). The top 50 returns in each search engine as well as the online index of mental health resources previously cited were screened according to the following inclusion criteria: (1) all consumer commentary was viewable without requiring registration or membership conditioned on moderator approval, and (2) the website contained at least 200 consumer comments for each drug. This search resulted in the consumer-generated websites www.askapatient.com and www.crazymeds.us. The former website contains pre-defined fields for users to input a 1 to 5 numerical rating of their satisfaction of the drug as well as their diagnosis, drug side effects, open-ended comments, sex, age, time taken, and dosage. The drug reviews then accumulate in a tabular format with little additional content on the website. The latter website hosts a discussion forum in which conversation threads are structured according to drug class and brand name. Neither website is monitored or edited by medical or health professionals, nor are postings edited for any reasons other than inappropriate content (ie, vulgar language or threats of self harm).

Professionally controlled commercial health websites (hereinafter referred to as “professionally controlled websites”) take the form of information portals monitored by health professionals and intended for a general audience of consumers and clinicians seeking a broad variety of online health information. The key criterion used in this study to identify professionally controlled websites was the oversight of content by a team of medical professionals, usually medical journalists who gather and write the content and medical doctors who provide oversight and consultation. Other inclusion criteria were: (1) the website was a commercial health portal (not operated by a governmental group or organization), (2) the given disclosures provided no evidence that the selected websites were owned by the same company or that they shared professional contributors, and (3) the website had received accolades for excellence in providing online health content. Governmental websites were excluded based on the reasoning that commercial health sites might be more likely to make concerted efforts toward assessing and adjusting website structure, functionality, and content in order to appeal to a broad and general audience and thereby increase site traffic. Google and Yahoo search engines were used to identify 2 professionally controlled health websites that were listed among the top 20 returns in searches of drugs’ brand names and met all inclusion criteria. This sampling resulted in the websites www.webmd.com and www.revolutionhealth.com. Content in both websites is pulled from a network of partners, including clinics, other health news sources, and health publishers, with oversight provided by health professionals and medical writers. Both websites are highly trafficked and lauded as reputable resources for up-to-date, authoritative health and treatment information.

Both professionally controlled websites also provide space for consumers to post ratings and reviews of drug treatment experiences. On WebMD, consumers are prompted to share a numerical 1 to 5 rating on the effectiveness, ease of use, and satisfaction of the drug, their diagnosis, age range, sex, how long they have taken the drug, and an open-ended comment. On RevolutionHealth, consumers are prompted to share a 1 to 10 rating on the effectiveness, ease of use, tolerability, and recommendation for the drug, their diagnosis, and an open-ended comment.

### Case Sampling

Escitalopram and quetiapine were selected as the points of entry for this study because both were top-selling drugs in the antidepressant and antipsychotic classes, respectively, at the time of this research [25-28]. Escitalopram was first approved by the US Food and Drug Administration (FDA) in 2002 for the treatment of depression and since 2006 has consistently earned an average US $2.5 billion in annual US retail sales. Quetiapine was first approved by the FDA in 1997 for the treatment of schizophrenia and was ranked as the ninth best-selling drug in 2006 with US $3 billion in US retail sales. It has maintained and exceeded that level of revenue in more recent years. Both drugs are also commonly used for numerous off-label purposes, including developmental disorders, anxiety, depression, and insomnia for quetiapine, and panic, social anxiety, premenstrual dysphoric disorder, and migraines for escitalopram [29].

All consumer reviews and commentary about the 2 drugs from the 4 websites through the end of February 2009 were imported into QDA Miner 3.2 data analysis software (see Multimedia Appendix 1) [30]. Each individual consumer was considered a single case. The comparison group of professional medication descriptions was retrieved from the 2 professionally controlled websites by importing all main text (excluding advertisements) returned from a search of the medications into QDA Miner 3.2 software. On WebMD, this text included the professionally controlled information on drug warnings, uses, side effects, precautions, interactions, and overdose. On RevolutionHealth, it included drug uses, side effects, dosage, interactions, and a section titled “Important information.”

Data collection resulted in a sampling frame of 6998 consumer cases (see Table 1) and the professional medication descriptions (all text for 2 medications on 2 websites). A stratified simple random sample of 120 consumer cases per drug per website (13.7% of the sampling frame) resulted in a coding sample of 960 cases (escitalopram, n = 480; quetiapine, n = 480). Since the sampling frame was not evenly distributed across websites, as illustrated in Table 1, this sampling strategy had the effect of oversampling consumer reviews on consumer-generated websites. Equal representation of consumer reviews from each website was thus ensured, and the coding sample became more manageable in size. All 4 professional medication descriptions were included in the analysis. 

Online consumer reviews were regarded in this study to be part of the public domain [31], and no personally identifiable information was collected. The Florida International University Office of Research Integrity approved this study.

**Table 1:** Website description and sampling frame for consumer reviews

			N Consumer Reviews	
Description		Selected Websites	Escitalopram	Quetiapine
**Professionally controlled commercial health websites**				
	Created and monitored by health professionals Reflect recognized standards of scientific/medical excellence Intended for lay and professional audiences May include pages where site users review and rate treatments	www.webmd.com^a^	1402	722
		www.revolutionhealth.com^a^	1873	624
**Consumer-generated health websites**				
	Not monitored or edited by medical or health professionals Contain only or mostly consumer-generated contributions (but may display some ads) Include ≥ 200 consumer reviews for each of escitalopram and quetiapine	www.askapatient.com	1093	791
		www.crazymeds.us	266	227
		Total	4634	2364

^a^The comparison group of professional medication descriptions was retrieved by copying all textual drug information returned from searches of each medication on the professionally controlled commercial health websites.

### Coding

**Figure 1:**
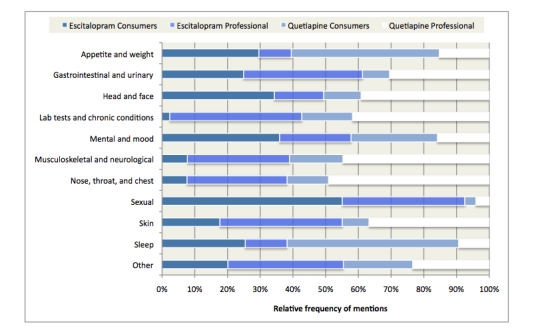
Relative frequency of mentions of effects in consumer reviews and in professional medication descriptions according to effect category

Author SH developed a codebook by inductively coding 85 randomly selected consumer cases from the sampling frame using initial and focused coding procedures [32]. This strategy was selected because a primary research aim was to explore consumer medication reviews on their own terms rather than fit them into a standardized vocabulary. Initial coding aimed to capture and condense literal meanings of reported medication effects with as little interpretation as possible. In keeping with the grounded theory approach, consumer text language was preserved. For example, descriptions such as “extreme sleepiness” were used as code names instead of the standard professional codes *drowsiness* or *somnolence*. Next, focused coding involved refining the initial codes to develop more definitive effect categories. Constant comparisons of data to data were used to ensure consistency in grouping drug effects. The final codebook identified 70 drug effects in consumer and professional text (eg, low libido, increased libido, trouble achieving orgasm) grouped into 11 effect categories (eg, sexual effects*,* see Figure 1)*.* The present analysis describes the 5 most frequently reported drug effects.

### Coding Agreement Analysis

A coding agreement analysis was conducted by author SH and another independent coder on 191 (20%) randomly selected cases. Intercoder agreement was calculated in QDA Miner 3.2 for each effect category on the level of code occurrence within a case using Scott’s pi (≥ .70 prespecified to indicate acceptable intercoder agreement) [33]. Both coders coded the first 100 cases and a Scott’s pi was calculated. The coders together reviewed each disagreement and came to a mutual decision about its resolution. After discussing individual coding decisions, the coders agreed upon collapsing or splitting some codes. The process was repeated with the next 91 cases. Author SH then coded the remaining 769 cases in the sample.

### Data Analysis

Frequency tables summarized consumer-reported drug effects. To compare consumer-reported effects and professional medication descriptions, we estimated the relative attention each group gave to specific effects by calculating the proportion of mentions of an effect out of all mentions of effects. Chi-square was calculated to test the null hypothesis of no association between website origination and consumer-reported drug effects. Significance tests were two-tailed and corrections were made for multiple comparisons by dividing the alpha level of .05 by k number of comparisons. Excerpts from text were extracted to illustrate differences in descriptions between consumer-generated and professionally controlled text.

An online health seeker is likely to visit only a few pages from each of 2 to 5 websites when researching health information online [3]. Therefore, the systematic evaluation of hundreds of consumer reviews performed in the present analysis has limited relevance to the everyday use of online consumer reviews for making treatment decisions. To simulate how a typical Internet user might consult consumer reviews while searching for medication-related information, then, the most recent 20 consumer comments on each drug from each website (n = 80) were compared for representativeness to all remaining consumer comments on that drug (n = 400). Chi-square was calculated to test the null hypothesis of no difference between recent and all remaining comments.

## Results

### Consumer Characteristics

Most consumers on AskaPatient and WebMD reported their gender, age, and length of time on the drug, while most consumers on the remaining 2 websites did not report gender or age, and at least half did not report length of time on the drug. Table 2 provides demographic characteristics for the consumers in this sample according to website on which the comment was posted. Table 3 shows the same information according to medication.

**Table 2:** Consumer characteristics according to website

		AskaPatient	CrazyMeds	WebMD	Revolution-Health	Total
Total n in each sample		240	240	240	240	960
Characteristics		n (%)	n (%)	n (%)	n (%)	n (%)
**Gender**						
	Female	158 (66)	64 (27)	179 (75)	22 (9)	423 (44)
	Male	81 (34)	27 (11)	50 (21)	10 (4)	168 (17.5)
	Not given	1 (< 1)	149 (62)	11 (5)	208 (87)	369 (38.4)
**Age in years**						
	≤ 18	15 (6)	3 (1)	5 (2)	2 (1)	25 (2.6)
	19–34	116 (48)	3 (1)	82 (34)	3 (1)	204 (21.3)
	35–54	93 (39)	10 (4)	103 (43)	6 (3)	212 (22.1)
	≥ 55	13 (5)	2 (1)	36 (15)	1 (<1)	52 (5.4)
	Not given	3 (1)	224 (93)	14 (6)	228 (95)	469 (48.9)
**Length of time on drug**						
	< 1 month	67 (28)	23 (10)	51 (21)	43 (18)	184 (19.2)
	1–6 months	72 (30)	38 (16)	59 (25)	42 (18)	211 (22)
	6 months–2 years	58 (24)	37 (15)	55 (23)	17 (7)	167 (17.4)
	≥ 2 years	41 (17)	24 (10)	56 (23)	10 (4)	134 (15)
	Not given	1 (<1)	120 (50)	17 (7)	128 (53)	266 (27.7)

**Table 3:** Consumer characteristics according to drug

		Escitalopram	Quetiapine
Total n in each sample		480	480
Characteristics		n (%)	n (%)
**Gender**			
	Female	216 (45)	207 (43.1)
	Male	77 (16)	91 (19)
	Not given	187 (39)	182 (37.9)
**Age in years**			
	≤ 18	7 (1.5)	18 (3.8)
	19–34	102 (21.2)	101 (21.2)
	35–54	113 (24)	99 (20.7)
	≥ 55	26 (4.4)	26 (5)
	Not given	232 (48.3)	235 (49)
**Length of time on drug**			
	< 1 month	106 (22.1)	78 (16.2)
	1–6 months	122 (25.4)	89 (18.5)
	6 months–2 years	85 (17.6)	82 (16.9)
	≥ 2 years	55 (11.5)	79 (14.4)
	Not given	112 (23.3)	152 (31.7)

### Intercoder Agreement

Table 4 shows that acceptable overall intercoder agreement was obtained (average Scott’s pi for all categories = .90 in phase 1, .82 in phase 2). Only for the category of *other effects* was agreement clearly unsatisfactory (< .41) because the miscellaneous effects included in it were grouped only after a substantial amount of coding had been completed.

**Table 4:** Intercoder agreement results

Drug Effect Categories	Scott’s Pi, Part 1 n = 100	Scott’s Pi, Part 2 n = 91
Appetite and weight	1	.84
Gastrointestinal and urinary	.88	1
Head and face	.82	.67
Lab tests and chronic conditions	1	.88
Mental and mood	.94	.74
Musculoskeletal and neurological	.82	.80
Nose, throat, and chest	1	.80
Sexual	.95	1
Skin	1	.65
Sleep	.93	.87
Other	.41	.33
Average overall	.90	.82

### Consumer Reported Effects

The most frequently mentioned effects by the 480 sampled consumers taking each drug were related to symptom improvement or worsening, and changes in sleep, weight, and appetite (see Multimedia Appendix 1). About one-fifth of escitalopram consumers also reported sexual effects. Approximately 30% (146/480) of consumers taking escitalopram and 25% (119/480) taking quetiapine reported an improvement in anxiety, depression, mania, or other symptoms. Another 15.8% (76/480) of consumers taking escitalopram and 10.2% (49/480) taking quetiapine reported new or worsening symptoms as an effect of the medication, including new or worsened panic attacks, depression, mania, or hallucinations. Significantly more consumers posting medication reviews on the consumer-generated (AskAPatient and CrazyMeds) than on the professionally controlled websites (WebMD and RevolutionHealth) reported quetiapine worsened their symptoms (17.3% or 38/220 versus 5% or 11/220, *P <* .001, significance level set at .002 for k = 25 comparisons), while more consumers posting reviews on professionally controlled sites reported it improved their symptoms (32.7% or 72/220 versus 21.4% or 47/220 *P* = .008). This trend held for escitalopram without reaching statistical significance.

Over 60% (291) of the 480 consumers taking quetiapine reported effects on sleep, with 35.6% (171) commenting the medication helped their sleep and 33.1% (159) that it caused excessive sleep and tiredness. For the 480 consumers taking escitalopram, sleep changes indicating excessive sleep were mentioned by 23.8% (114), while 13.3% (64) of consumers mentioned insomnia. For quetiapine, over 30% (148) of consumers reported a range of appetite and weight effects, most notably weight gain (22.5% or 108/480). For escitalopram, 13.1% (63) of consumers reported weight gain and 4.8% (23) weight loss. Finally, 20.2% (97) of consumers taking escitalopram reported sexual effects, primarily in the form of low libido (10.6% or 51/480) and trouble achieving orgasm (8.5% or 41/480).

### Consumer Reported Effects Compared With Professional Medication Descriptions

**Figure 2:**
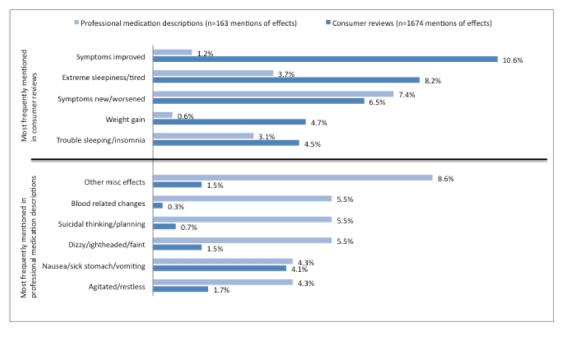
Most frequently mentioned effects as a proportion of all mentions of escitalopram effects in consumer-generated and in professionally controlled text

Figure 1 lists the relative frequency of mentions of drug effects in consumer reviews and professional medication descriptions across the 11 effect categories, and Figure 2 compares consumer reviews and professional descriptions on the most frequently mentioned effects for each drug as a proportion of all mentions of effects in each of the respective texts. For both medications, professional descriptions on WebMD and RevolutionHealth frequently mentioned worsening mental or mood effects, such as agitation and suicidal thinking, as well as physical effects, including dizziness, weakness, and vision problems. Other miscellaneous effects (such as toothache or bronchitis) were also more frequently mentioned in professional descriptions from both websites compared with consumer reviews.

**Figure 3:**
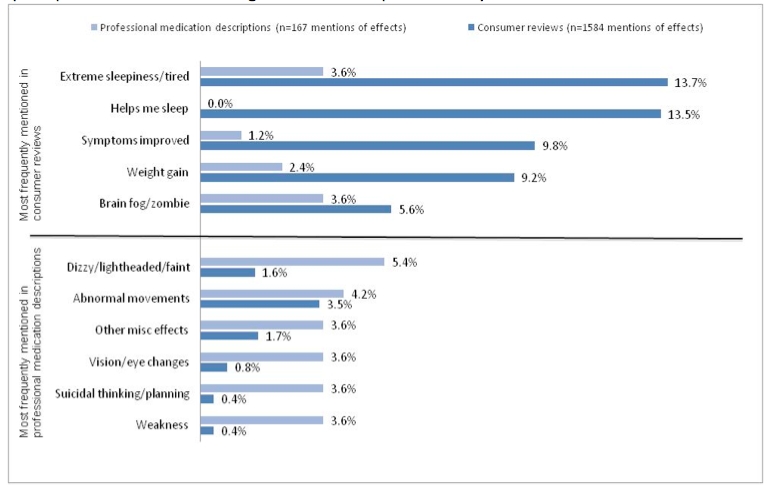
Most frequently mentioned effects as a proportion of all mentions of quetiapine effects in consumer-generated and in professionally controlled text

The following three tables illustrate qualitative differences between consumer-generated and professionally controlled text. Table 5 compares website text with respect to worsening symptoms while taking escitalopram. Listed are the standard warnings in professional medication descriptions for worsened symptoms and suicidality while taking antidepressants (seemingly derived from the FDA-approved drug label) in addition to consumer reviews that identify the same effects but further illustrate their various manifestations.

**Table 5:** Worsening symptoms on escitalopram according to consumer-generated and professionally controlled text

Website	Standard Warning^a^ (Professionally Controlled Text)	Consumer Review^b^ (Consumer-Generated Text)
RevolutionHealth	*Call your doctor at once if you have any new or worsening symptoms such as mood or behavior changes, anxiety, panic attacks, trouble sleeping, or if you feel impulsive, irritable, agitated, hostile, aggressive, restless, hyperactive (mentally or physically), more depressed, or have thoughts about suicide or hurting yourself*	*A couple of days later I had my first manic experience which lasted about 30 minutes of complete reckless driving, I probably should have gotten arrested. And a few minutes later I came down into deep depression*. [Consumer review #258]
WebMD	*Tell the doctor immediately if you notice worsening depression/other psychiatric conditions, unusual behavior changes (including possible suicidal thoughts/attempts), or other mental/mood changes (including new/worsening anxiety, panic attacks, trouble sleeping, irritability, hostile/angry feelings, impulsive actions, severe restlessness, very rapid speech)*	*I have been very hostile and irritable on this med and my panic attacks have been coming more often and they have been much worse! I have no patience with my kids or my fiancé, or basically anyone around me.* [Consumer review #364]
AskaPatient		…[a]*nd then the worst crippling panic attacks I have ever had to date*… [Consumer review #8] *I seemed to become more aggressive and assertive. I would just speak my mind whenever I got angry, and had no fear. I seemed to become more “mean” and “mad” and I just didn’t like myself*. [Consumer review #41]
CrazyMeds		*Had some hypomania then extreme agitation, then suicidality. The agitation was awful, felt like I was going to jump out of my skin— and my mind was racing.* [Consumer review #172] …*2 hours of alternating panic attacks/crying jags…* [Consumer review #130]

^a^ Complete text is provided.

^b^ Selected illustrative comments are provided

Table 6 shows that most of the mentions of sexual effects of escitalopram in professional medication descriptions were related to *other* sexual effects, such as the nondescript sexual problems and priapism. Of all mentions of sexual effects, consumers most frequently discussed lost sex drive (42.2%) and trouble achieving orgasm (37%), though the former was described in the professionally controlled text on WebMD as infrequent and was absent from RevolutionHealth. Professional descriptions used the terms *less serious*, *less severe*, or *severe* to describe sexual effects, while consumers consistently described these as “the absolute worst,” or “extremely frustrating,” and made comments such as, “I want to quit…so I can have a frigging orgasm” or “can’t perform sexually so you get depressed and anxious.”

**Table 6:** Sexual effects of escitalopram according to consumer-generated and professionally controlled text

	Mentions of Sexual Effects		Professional Medication Descriptions of Sexual Effects		Consumer Reviews
	Professional Descriptions	Consumer Reviews	WebMD^b^	Revolution-Health^b^	All Websites^c^
Total n	9	135			
Sexual effect	n (%)	n (%)			
Lost sex drive	2 (22.2)	57 (42.2)	Unlikely but serious	--	“very bothersome”
Trouble achieving orgasm	3 (33.3)	50 (37)	Common, less severe	Less serious	“the absolute worst” “extremely frustrating”
Other sexual effects^a^	4 (44.4)	16 (11.9)	Infrequent, less severe	--	“significant sexual effects”

^a^In professionally controlled text, this code included only the terms *priapism* and *sexual dysfunction*. In consumer reviews, this code included the terms *sexual side effects*, *sexual dysfunction,* and *sexual problems*.

^b^Complete text is provided.

^c^Illustrative comments are provided.

Table 7 illustrates notable qualitative differences also observed in the described sleep effects of quetiapine. While approximately one-third of consumers reported the drug helped them sleep, this benefit was absent from professional medication descriptions, which only mentioned drowsiness or tiredness as a “less severe” side effect of quetiapine. The typical excerpts from consumer reviews listed in Table 7 describe the sleep effect as sometimes helpful and sometimes burdensome, depending on the individual’s circumstances and needs at the time.

**Table 7:** Sleep effects of quetiapine according to consumer-generated and professionally controlled text

Website	Sleep Effect^a^ (Professionally Controlled Text)	Sleep Effect^b^ (Consumer-Generated Text)
RevolutionHealth	*The following warnings are available for this medication…may cause drowsiness*	*[It] puts me to sleep. It’s that simple. I take it and within an hour I’m out—unwakable—for the next 12 or more hours.* [Consumer review #739] …*helped very, very effectively with sleep: 30 minutes max after taking 125-150 mg at night, I am out for good.* [Consumer review #808] …*the worst side effect is the sleepiness—I sleep 10-12 hours a day and still have periods when I have to nap (or could fall asleep standing up).* [Consumer review #773]
WebMD	*Common side effects: drowsiness…less severe, tiredness*	*It helped me sleep very well, but I was very groggy in the morning* [Consumer review #871]
AskaPatient		*So while it does provide me sleep…it’s the kind of sleep that wouldn’t allow me to be woken, even if my house is on fire. I am not able to be woken from this coma-like sleep for hours. That scares me*. [Consumer review #515] *…extreme sleeping…* [Consumer review #570]
CrazyMeds		*I like what this drug does to me (sleepy bye bye land).* [Consumer review #172] *You’ll sleep until next Tuesday. Of course, that could be a good thing, depending on how your life is at this moment.* [Consumer review #1084]

^a^Complete text is provided.

^b^Selected illustrative comments are provided.

### Representativeness of Recent Consumer Reviews

Table 8 compares comments from the most recent 20 consumer reviews from each of the 4 websites to comments in all remaining 400 consumer reviews for each drug on effects mentioned by more than 10% of consumers. For all but 2 effects (extreme sleepiness/tired for escitalopram and brain fog/zombie for quetiapine), frequencies of reported effects in recent comments were quite comparable to frequencies in all remaining reviews.

**Table 8:** Twenty most recent consumer reviews compared with all remaining consumer reviews for each drug on each website for effects mentioned by more than 10% of consumers

	Escitalopram			Quetiapine		
Drug effects	80 Recent Reviews	Remaining 400 Reviews	*P* Value	80 Recent Reviews	Remaining 400 Reviews	*P* Value
	n %	n %		n %	n %	
Symptoms reduced/improved	26 (32.5)	145 (30.4)	.67	23 (28.8)	96 (24.8)	.37
Symptoms new/worsened	15 (18.8)	75 (15.8)	.43	9 (11.3)	40 (10.2)	.74
Extreme sleepiness/tired	26 (32.5)	113 (23.8)	.045	26 (32.5)	133 (33.1)	.90
Weight gain	11 (13.8)	62 (13.1)	.84	16 (20)	92 (22.5)	.56
Brain fog/zombie	10 (12.5)	51 (10.8)	.58	18 (22.5)	55 (15.2)	.047

## Discussion

### Principal Results

Online consumer-generated and professionally controlled text bearing on the same psychotropic drugs reported many of the same drug effects but differed substantially in their descriptions and in the relative frequency of mentions of certain effects. Consumers more frequently discussed effects with an obvious manifestation and immediate impact on their daily lives, such as excessive sleeping and weight gain. Other than repetitions of regulatory warnings about serious adverse mental or mood effects (increased suicidal ideation, for example), professional medication descriptions most often mentioned physical side effects, such as dizziness and vision problems. Additionally, descriptive labels applied in professional text, such as *less serious* or *severe*, rarely matched with the perceived importance or severity of common effects according to consumers. For example, *less severe drowsiness* caused by quetiapine, as described in professional text, can translate to “coma-like sleep” or having to miss work because of the inability to stay awake, as described in consumer reviews. 

Consumer reporting of medication effects also varied across health websites, with consumers posting reviews on professionally controlled health websites more often reporting greater symptom improvement, less symptom worsening, and fewer side effects. These differences may be partly explained by visual cues and normative themes present on websites that may attract drug consumers who share a particular perspective or attitude. For example, WebMD receives substantial revenue from pharmaceutical company sponsored advertisements, which may in turn attract users who hold a favorable disposition towards medication taking.

Finally, a cursory examination of only recent consumer comments on a particular medication, as might be viewed by the “typical” Internet user seeking online information from consumer-generated text, reflected commonly reported drug effects in proportion to a full representative sample of consumer reviews.

Overall, consumer-generated and professionally controlled medication descriptions each offer distinct advantages and disadvantages in helping to make treatment decisions or gauge the predictability of one’s personal medication experience. Professional medication descriptions on commercial health portals provide succinct and comprehensive summaries of possible effects, but the meaningfulness of this information is limited by the lack of context. Consumer reviews, on the other hand, provide abundant concrete descriptions and situational examples of how specific effects may manifest in various combinations and to varying degrees. While the lack of organization of consumer reviews—which are individually dispersed across many websites and sometimes quite numerous on a single website—limits their integration into coherent wholes for consumers and clinicians consulting them to aid treatment decisions, this research provides initial empirical evidence for the representativeness and usability of a typical brief browsing strategy involving consumer reviews.

Nevertheless, unless the online health searcher who uses consumer reviews actively seeks a variety of sources to retrieve consumer reviews, differences in reporting across websites—such as those observed in this study where professionally controlled websites contained more positive consumer comments and consumer-generated websites contained more negative comments—could unknowingly hinder informed decision-making. At the same time, if professional medication descriptions could more richly describe the range or impact of drug effects in ordinary situations and contexts, then online consumer reviews might not constitute such a necessary innovation for the many active and potential drug consumers who consult them. In the current environment, clinicians and consumers seeking medication information on the Internet may want to be open to consulting consumer-generated content but vigilant when reviewing it and are encouraged to maximize their exposure to a variety of drug accounts by utilizing a diversity of online consumer-generated and professionally controlled sources.

### Limitations

A major limitation to all research relying on Internet data is the inherent anonymity of online users. While the accuracy of self-report data is naturally a concern in all research designs, the anonymity of Internet users adds the possibility of data contributions from persons with vested interests. Pharmaceutical industry literature, for example, has expressed a clear interest in utilizing online patient communities to build brand trust [34]. No method exists to distinguish genuine from possibly unauthentic consumer accounts, and few studies have attempted to address this problem [35]. Despite unknown authenticity and credibility, however, consumer-generated health content is quickly gaining popularity and carries utility for its users, making its description an important initial step for continued research. The present study further found that consumer-generated data does correspond with professional medication descriptions, which may add validity to these anonymous consumer Internet postings.

Also, the present study did not explore differences in drug effects according to diagnosis, reason for use, or indication, partly due to inconsistent reporting of this information by online consumers. When this information was provided, it was further difficult to parcel out diagnosis (ie, bipolar disorder) from individuals’ stated reason for using a drug (ie, to help with sleep). With large proportions of consumers reporting, for example, sleep changes on quetiapine, it appears that some effects are experienced globally regardless of diagnosis or indication [36]. Further, most consumers (64%) did not report the dose of the drug they were taking, and many who did described trying multiple doses, which made it difficult to isolate any dose-effect relationships for the purposes of this analysis. Finally, while data collection strategies aimed to capture information on the immediate-release, brand-name versions of the two selected drugs, consumers may not have made the distinction in their reviews between brand name versus generic or immediate versus extended release. It is, therefore, possible that some consumer reviews described experiences of different versions of the selected medications.

Despite these limitations, this research used a mixed qualitative and quantitative analysis of a large representative sample of Internet data from a purposively varied selection of websites. All textual data thus obtained were submitted to coding. Three strategies to minimize interpretive biases in qualitative coding methods were used: the research grounded codes in the data by preserving consumers’ language in developing code names and categories, maintained utmost transparency by using tracking features in QDA Miner 3.2 software, and tested for the reliability of assigned codes by measuring agreement with a second independent coder.

### Comparison With Prior Work

Notable similarities and differences exist between consumer-reported effects in this sample and other estimates of drug effects. An online service that collects drug safety information from its patient community, iGuard.org, surveyed a random sample of 700 members taking 1 of 5 antidepressants, including escitalopram [37]. Congruent with the present findings, the most frequently reported side effects were sexual dysfunction (24.5%), sleepiness (23.1%), and weight gain (21.4%). The FDA-approved label for escitalopram lists lower rates of these effects, reporting that 1% to 7% of clinical trial participants with major depressive disorder and anxiety experienced decreased libido or impotence, and 6% to 13% experienced somnolence, while no clinically important changes in body weight were observed. Postmarketing studies of antidepressants have estimated higher but varying rates of sexual effects, affecting 20% to 80% of users [38-40]. Research on escitalopram-induced weight gain has shown the effect to be minor [41], and data on sleep show, as the present findings, both sedative and stimulant effects [42,43].

Similarly, for quetiapine, the FDA drug label cites 4% to 22% of participants in clinical trials experiencing weight gain, an effect mentioned by 22.5% of consumers in this study. Since the release of quetiapine on the US market in 2002, weight gain and metabolic disorders have been recognized as significant problems for all atypical antipsychotics, though quetiapine is typically regarded as causing less weight gain than other medications in its class [44-46]. Reports from consumers in this study also seem to reflect real-world use of quetiapine as a sleep aid, among other frequent off-label uses [47-49]. 

### Conclusions

If online consumer medication reviews can offer meaningful information to those contemplating or making treatment decisions, as this research suggests, then such reviews may further be useful for postmarketing safety surveillance. Current safety surveillance systems, such as the FDA’s MedWatch, are known to capture only a fragment of medically defined serious adverse events. The dispersion of consumer reviews within and across websites, their lack of a standardized vocabulary for reporting drug effects, and sparse detailing of the main elements of a conventional adverse event report currently limit their practical value for surveillance. Technology to integrate and organize in a searchable format the mass of dispersed consumer medication reviews may partially address these limitations and hold the potential to be an innovative addition to a currently deficient system [50]. In the meantime, informed discussion, creative suggestions, as well as guidance from the FDA regarding the responsibility of website owners and pharmaceutical companies over monitoring and reporting adverse events found in online consumer reviews and patient communities are needed [51].

The findings of this study suggest avenues for continued research. First, the present analysis could be replicated using a standardized medical coding vocabulary (ie, MedDRA) in order to facilitate comparison with other pharmacoepidemiological databases. The present analysis could also be replicated (1) to determine if online consumers report effects in similar proportion for additional medications and websites and (2) to search for temporal trends and patterns in types of effects reported and their associations with large-scale events such as warnings from regulatory agencies or direct-to-consumer ad campaigns for medications. Secondly, it is unclear if discrepancies in drug effects between consumer reviews and information derived from conventional drug research represent an overestimation of effects by online consumers or an underestimation of effects in drug research. To address this, controlled clinical trials could incorporate simple targeted measures for weight, sleep, and sexual effects, rather than continue to rely on spontaneous or unsolicited participant self-report for such data (a method that tends to underestimate the true frequency of events) [52,53]. Lastly, this research suggests that current strategies for filtering online health searches to return only *trusted* or *approved* websites [5,6] may inappropriately address the challenge to identify quality health sources on the Internet because such strategies unduly limit access to an entire complementary source for health information.

## Multimedia Appendix 1

Powerpoint presentation: Can online consumers contribute to drug knowledge and drug safety? An examination of consumer reporting of drug effects across health websites

## Abbreviations

FDA: Food and Drug Administration
