# Supplementary material for: Can Online Consumers Contribute to Drug Knowledge? A Mixed-Methods Comparison of Consumer-Generated and Professionally Controlled Psychotropic Medication Information on the Internet
Source: J Med Internet Res. 2011 Jul 29;13(3):e53. doi: 10.2196/jmir.1716 (PMC3222176; doi:10.2196/jmir.1716)
Supplement: Supplementary file 1 [file jmir_v13i3e53_app1.ppt]

## Slide 1
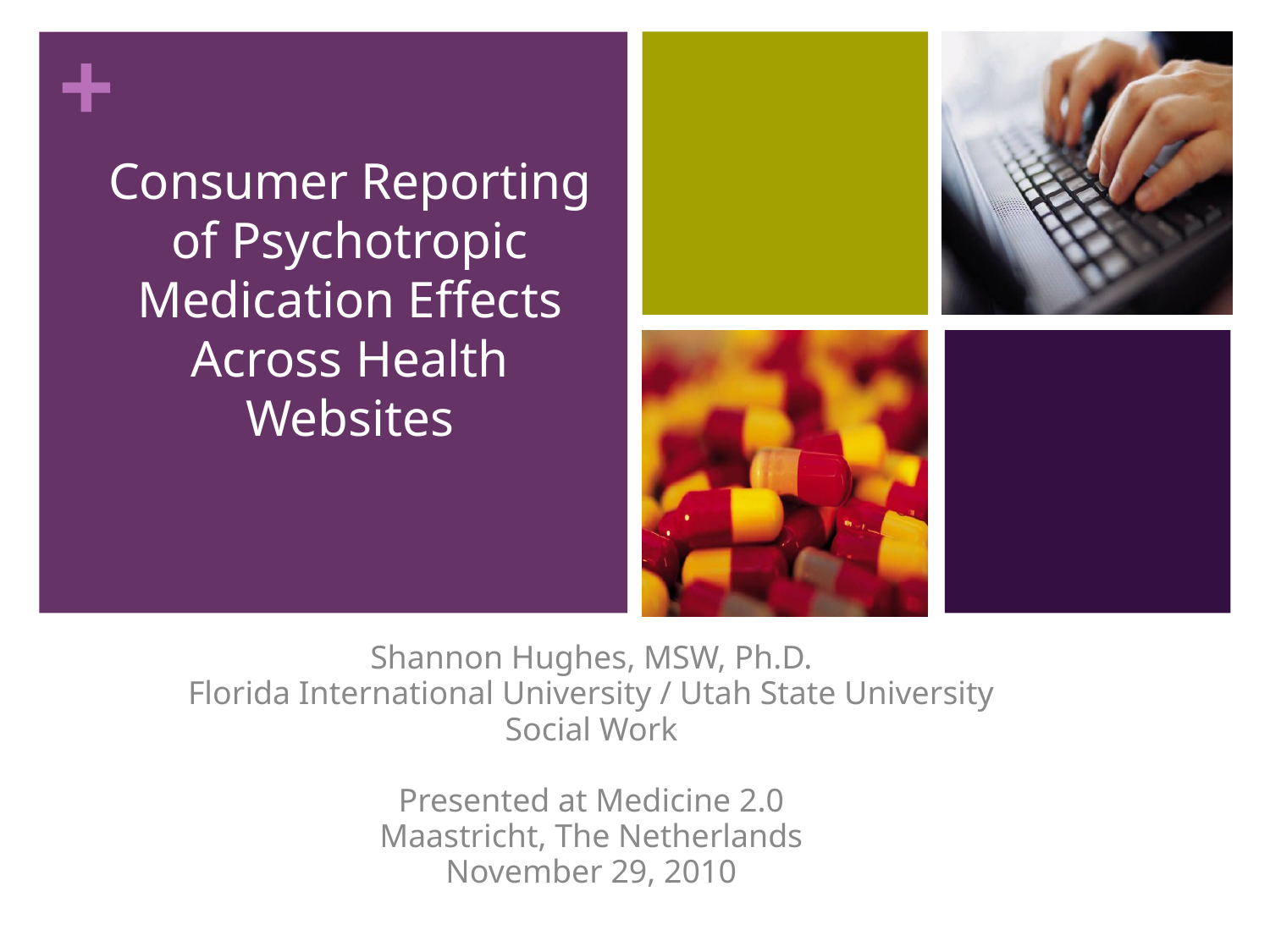

# Consumer Reporting of Psychotropic Medication Effects Across Health Websites
Shannon Hughes, MSW, Ph.D.
Florida International University / Utah State University
Social Work
Presented at Medicine 2.0
Maastricht, The Netherlands
November 29, 2010

## Slide 2
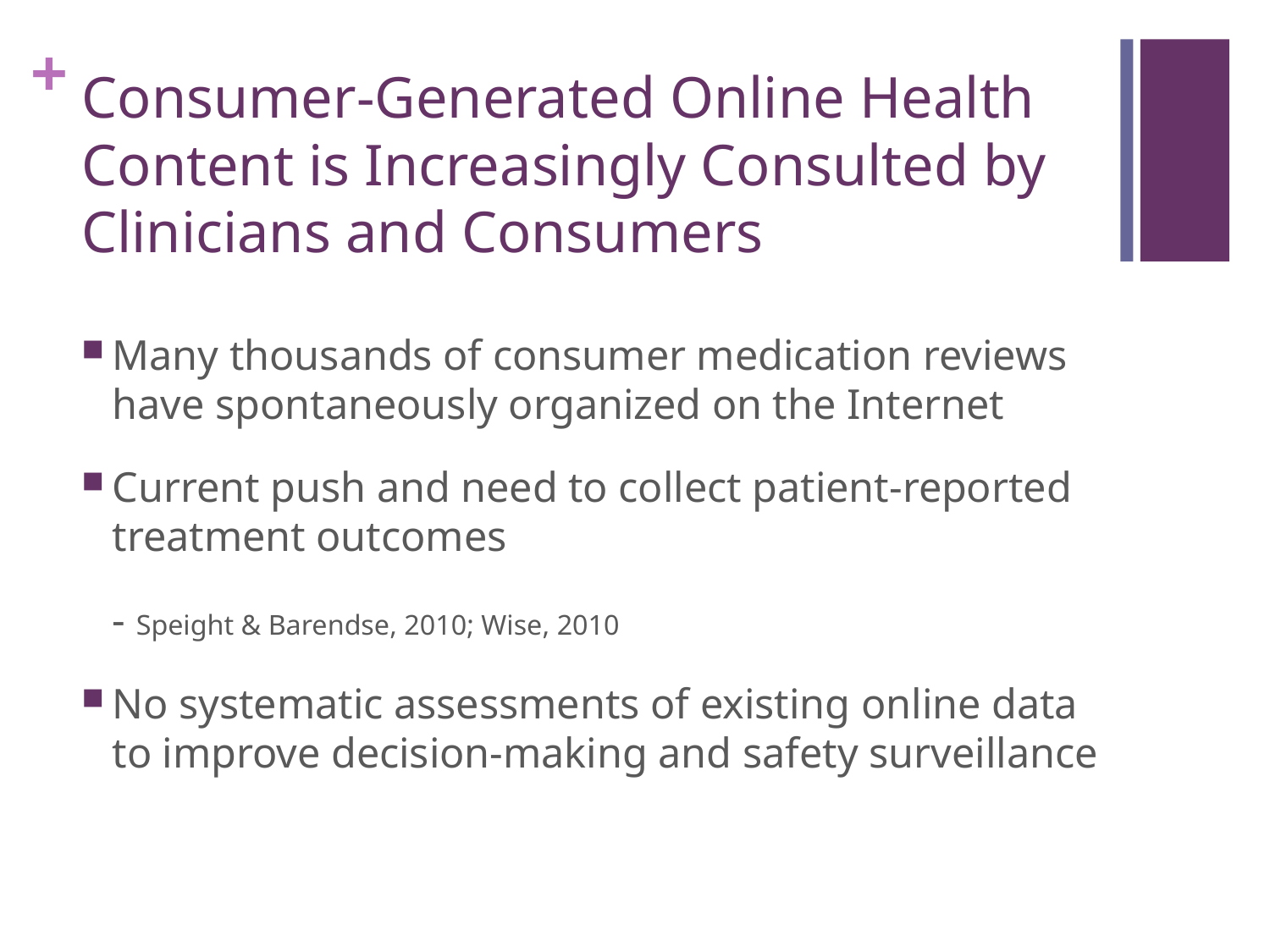

# Consumer-Generated Online Health Content is Increasingly Consulted by Clinicians and Consumers
Many thousands of consumer medication reviews have spontaneously organized on the Internet
Current push and need to collect patient-reported treatment outcomes
					- Speight & Barendse, 2010; Wise, 2010
No systematic assessments of existing online data to improve decision-making and safety surveillance

## Slide 3
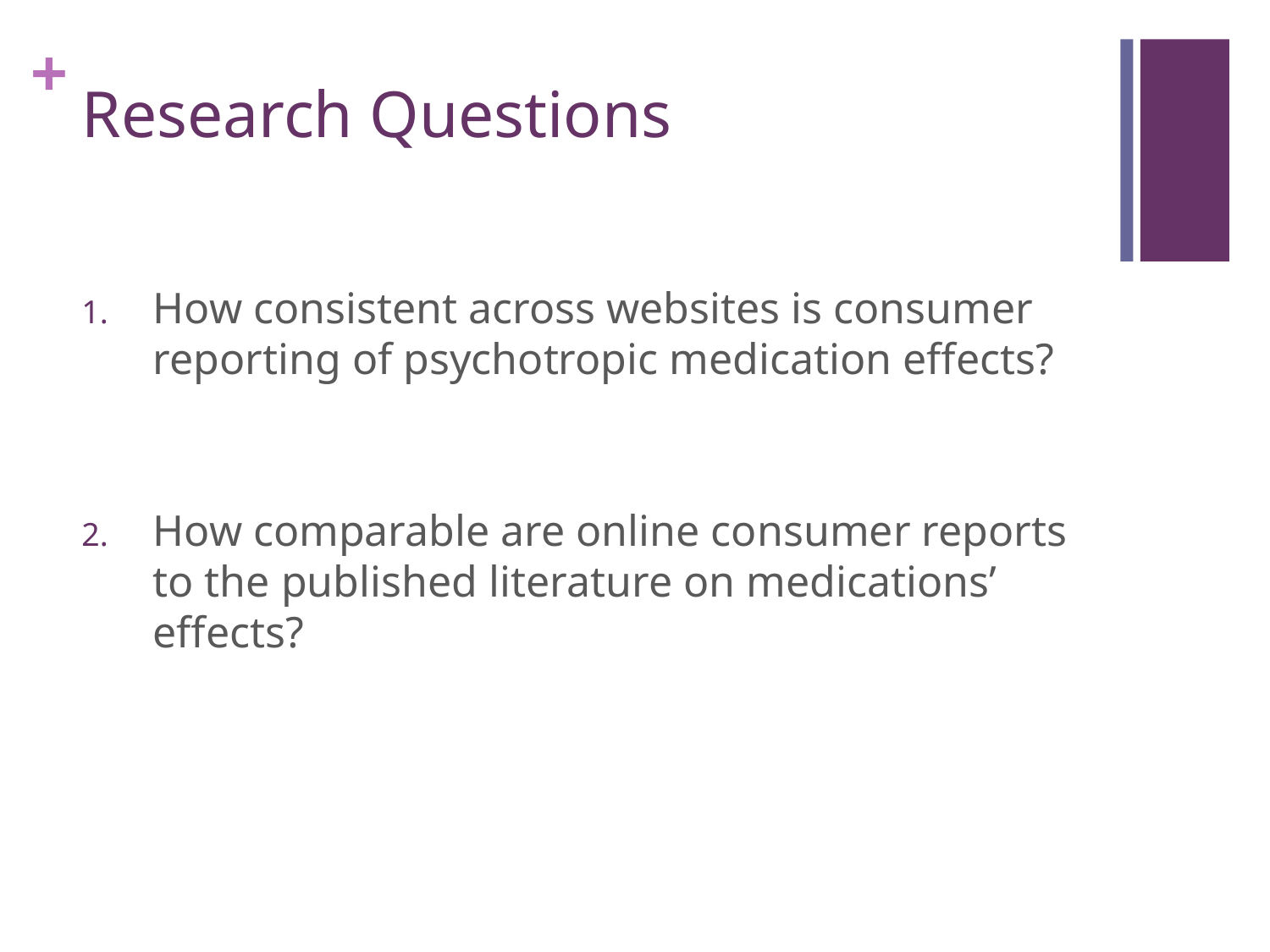

# Research Questions
How consistent across websites is consumer reporting of psychotropic medication effects?
How comparable are online consumer reports to the published literature on medications’ effects?

## Slide 4
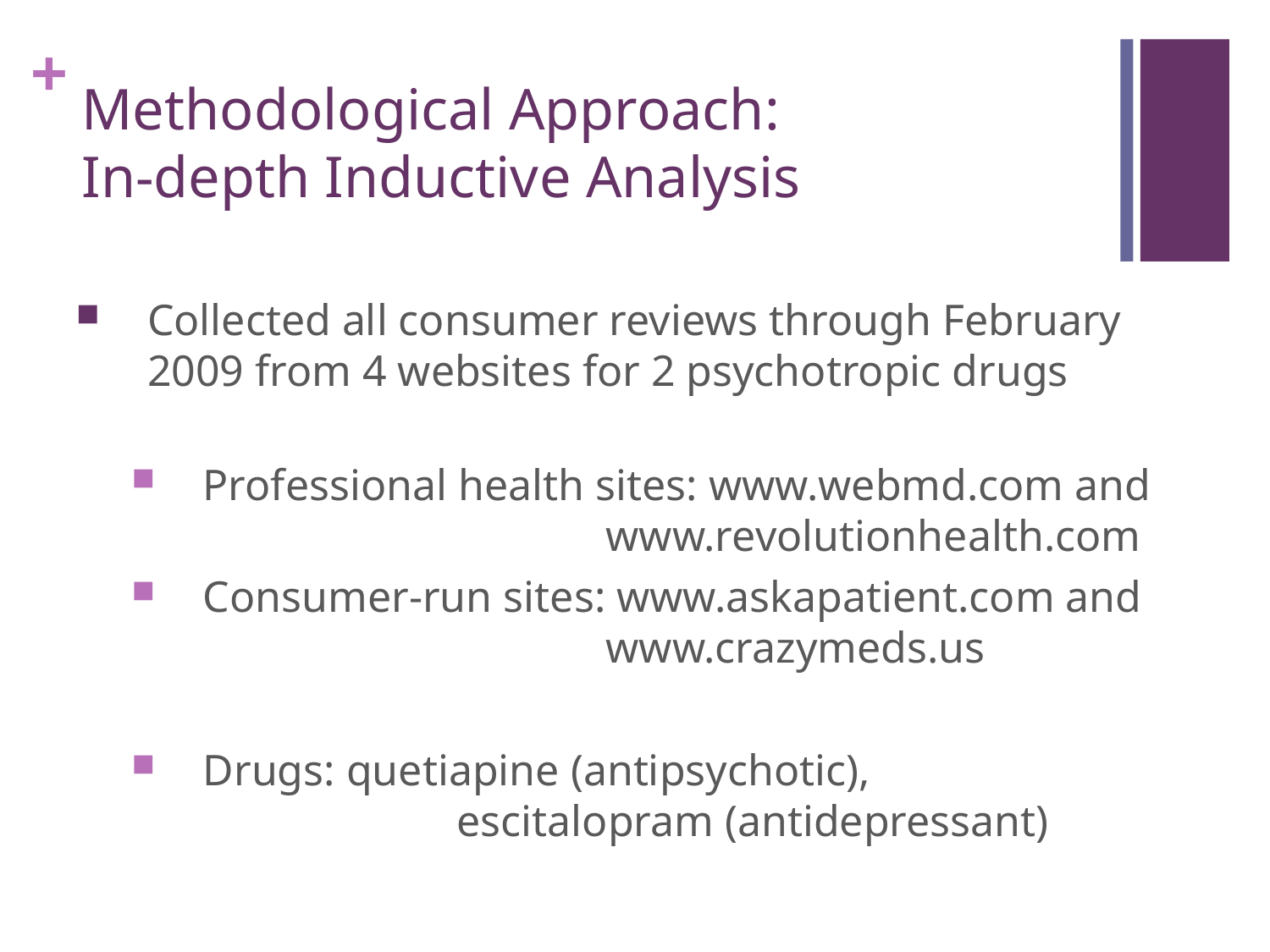

# Methodological Approach: In-depth Inductive Analysis
Collected all consumer reviews through February 2009 from 4 websites for 2 psychotropic drugs
Professional health sites: www.webmd.com and 			 www.revolutionhealth.com
Consumer-run sites: www.askapatient.com and 			 www.crazymeds.us
Drugs: quetiapine (antipsychotic),	 	 	 	escitalopram (antidepressant)

## Slide 5
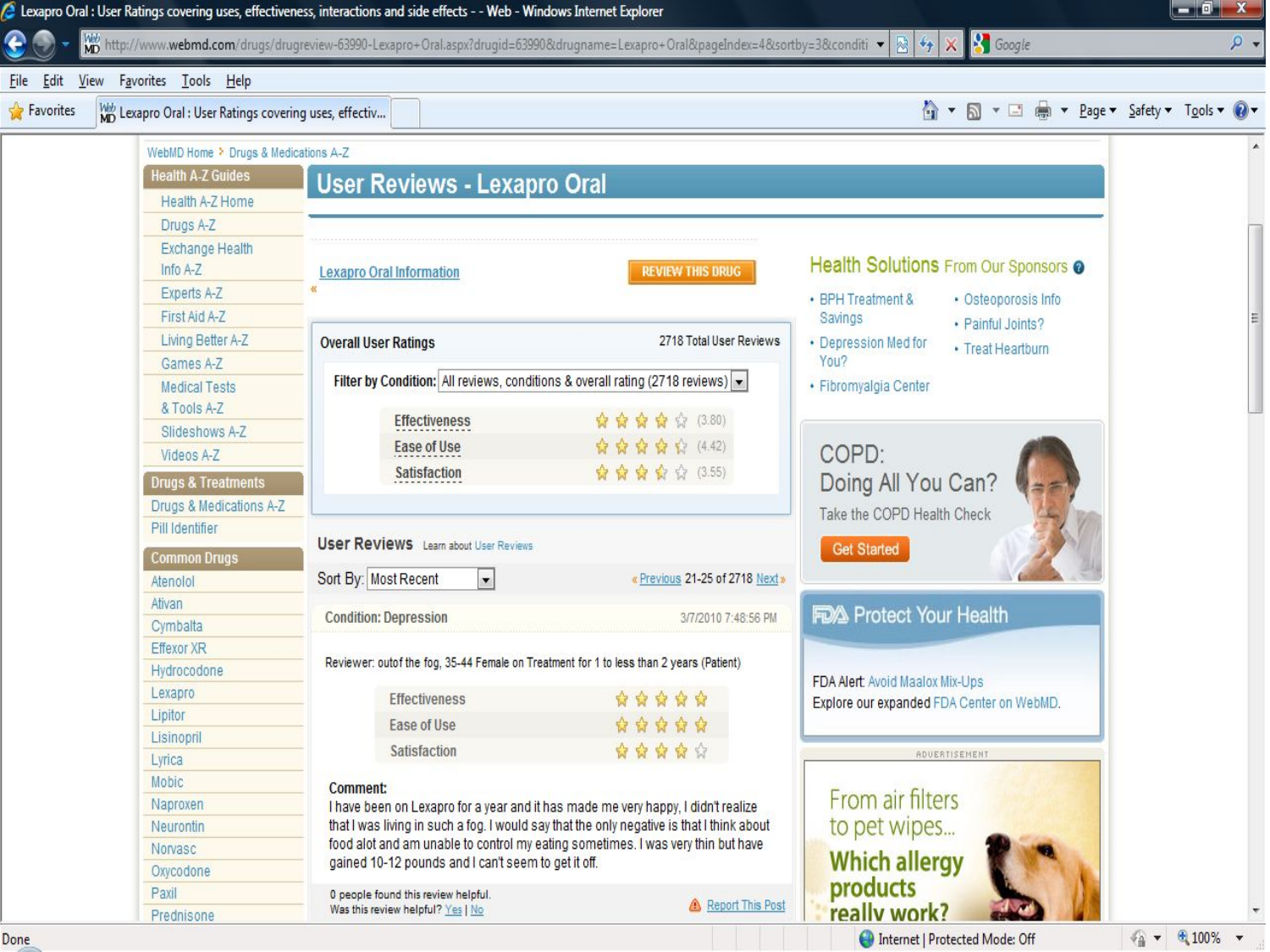

#

## Slide 6
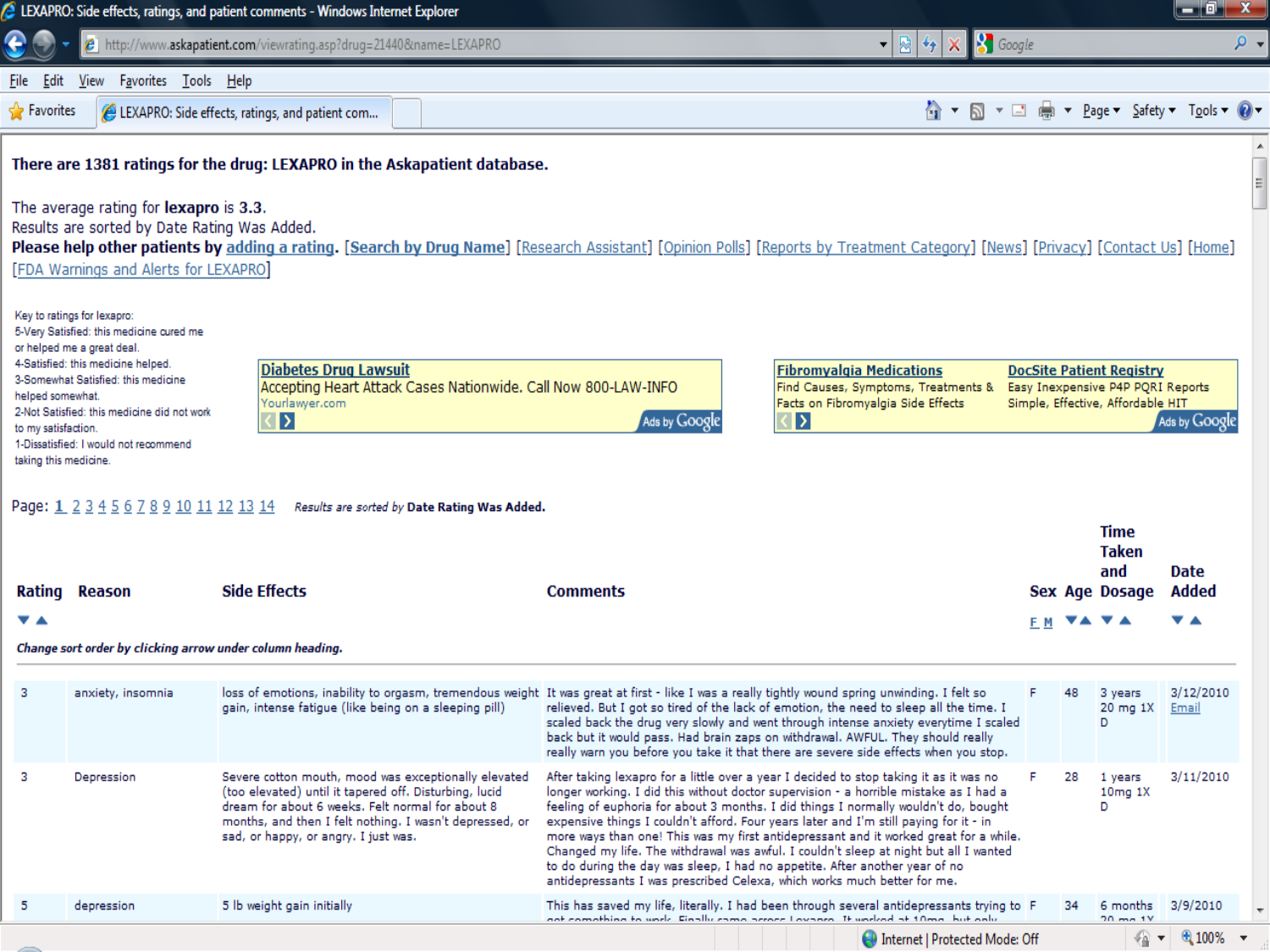

#

## Slide 7
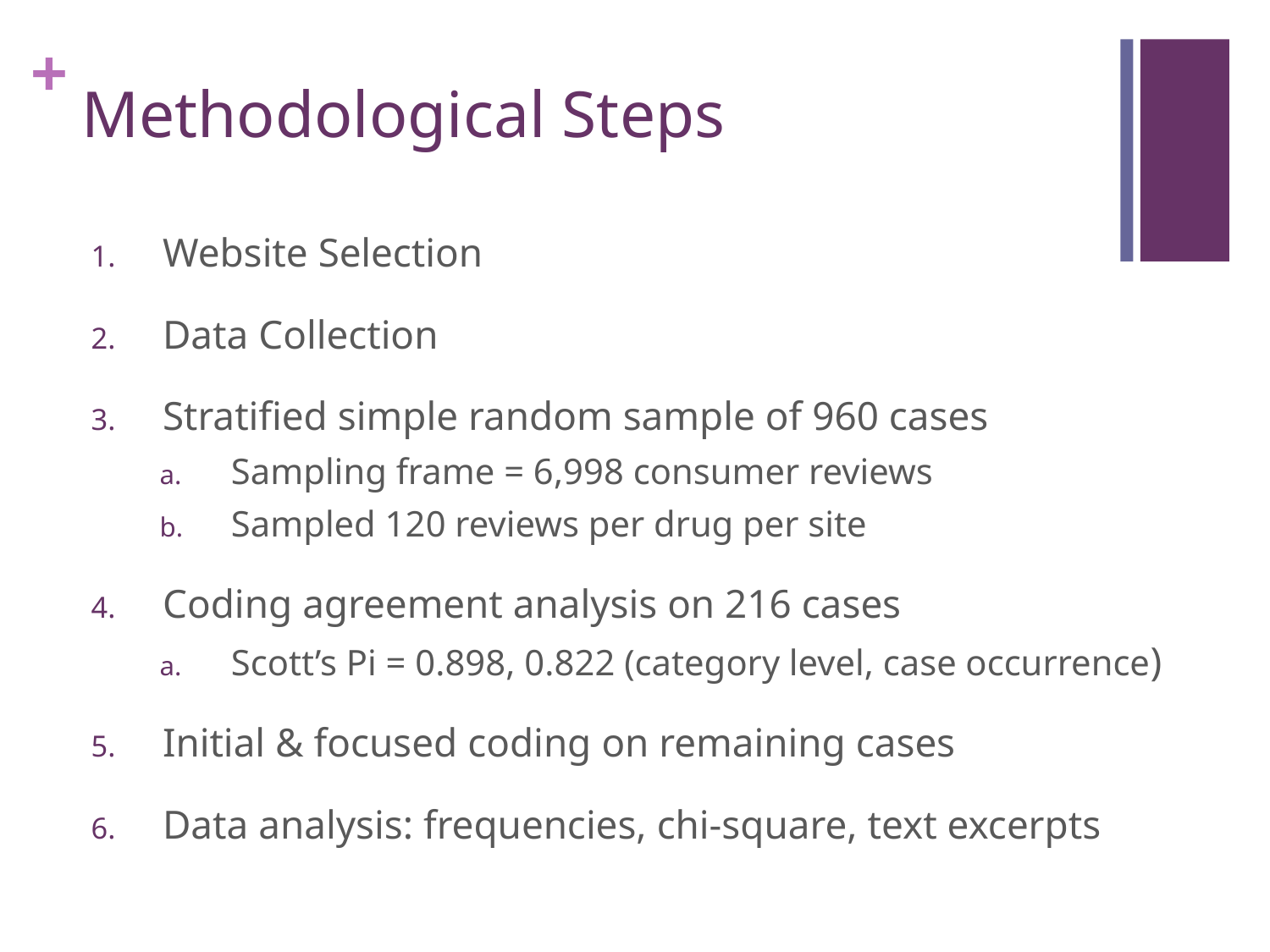

# Methodological Steps
Website Selection
Data Collection
Stratified simple random sample of 960 cases
Sampling frame = 6,998 consumer reviews
Sampled 120 reviews per drug per site
Coding agreement analysis on 216 cases
Scott’s Pi = 0.898, 0.822 (category level, case occurrence)
Initial & focused coding on remaining cases
Data analysis: frequencies, chi-square, text excerpts

## Slide 8
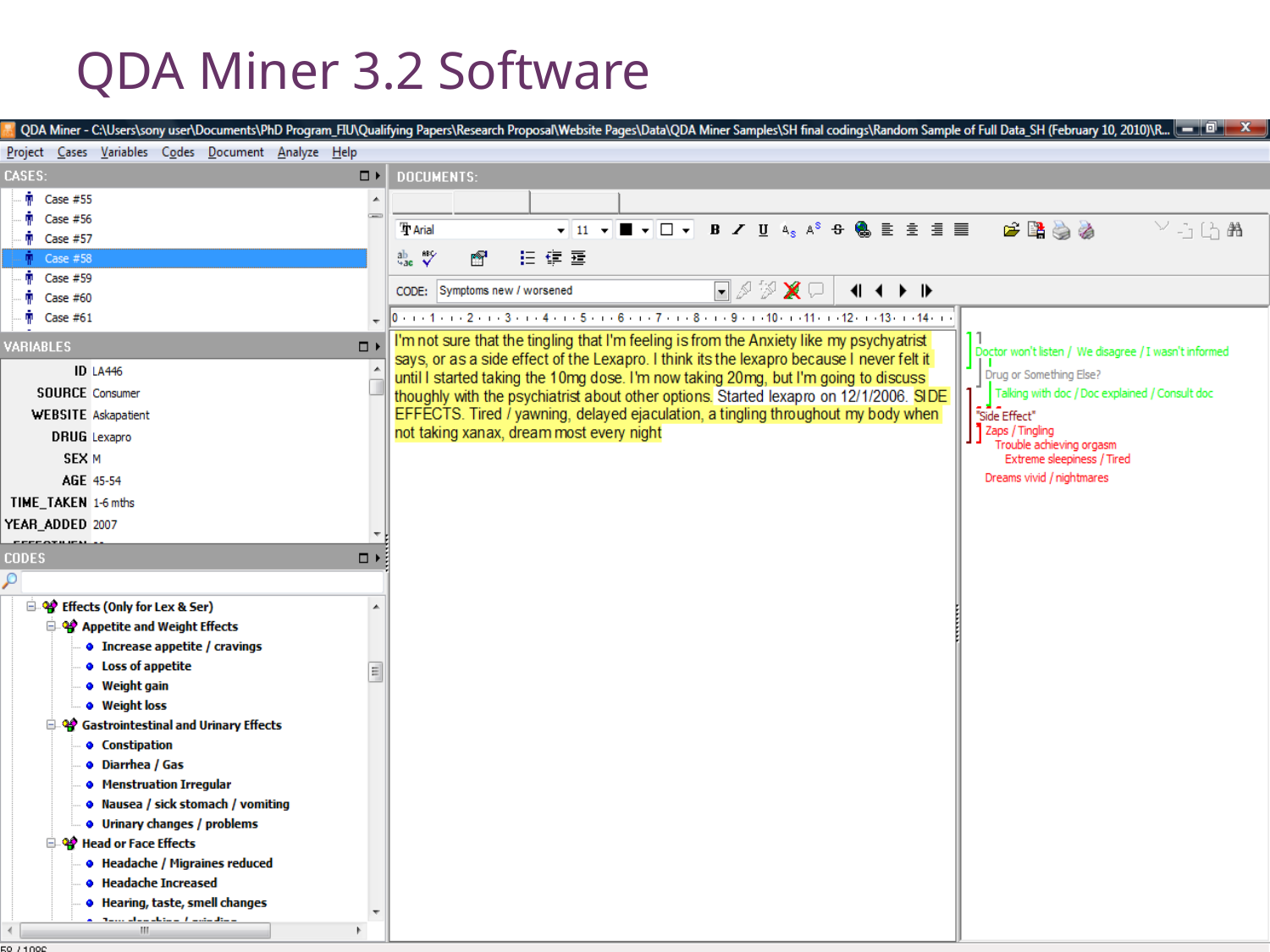

# QDA Miner 3.2 Software

## Slide 9
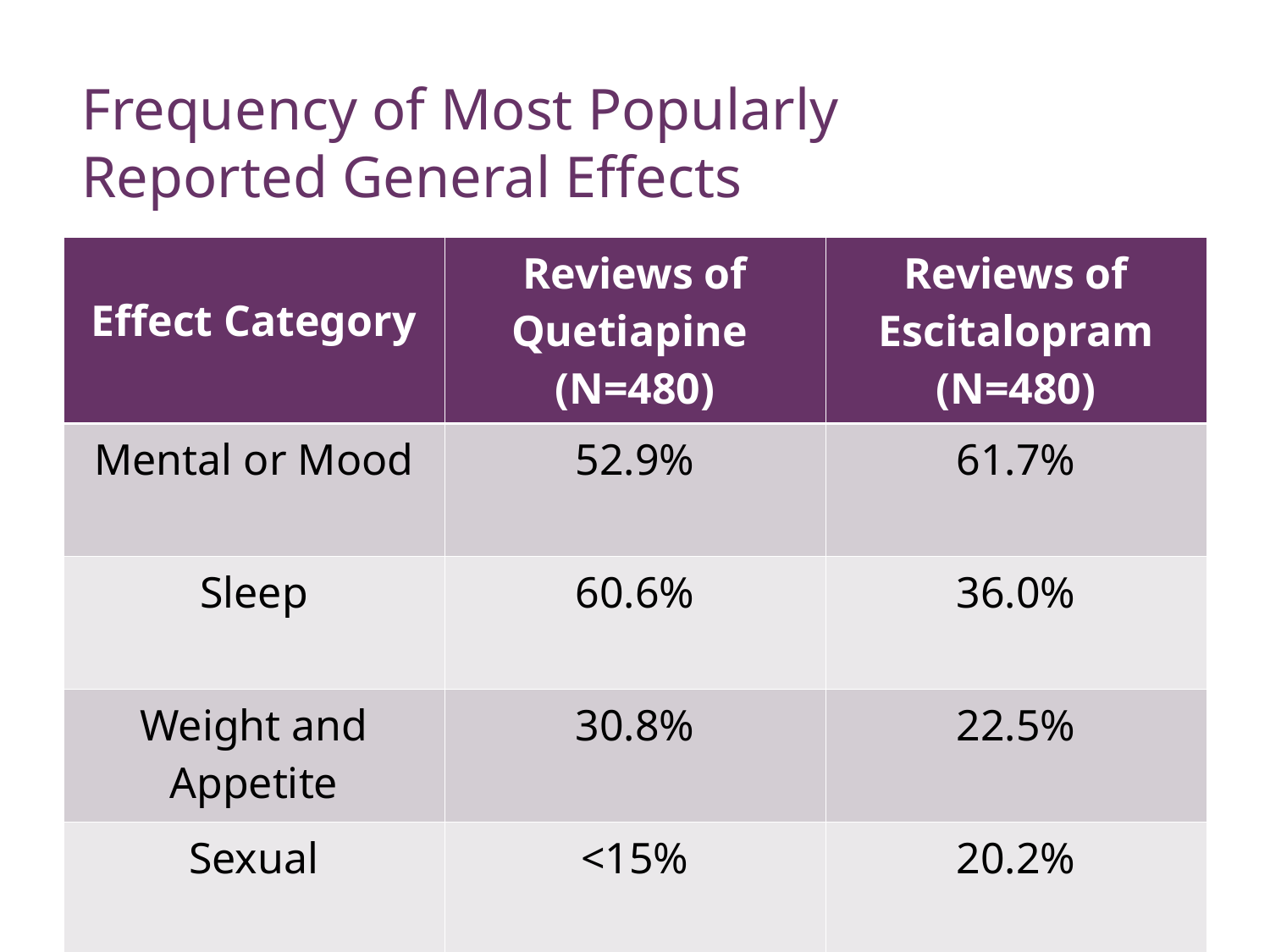

# Frequency of Most Popularly Reported General Effects
| Effect Category | Reviews of Quetiapine (N=480) | Reviews of Escitalopram (N=480) |
| --- | --- | --- |
| Mental or Mood | 52.9% | 61.7% |
| Sleep | 60.6% | 36.0% |
| Weight and Appetite | 30.8% | 22.5% |
| Sexual | <15% | 20.2% |

## Slide 10
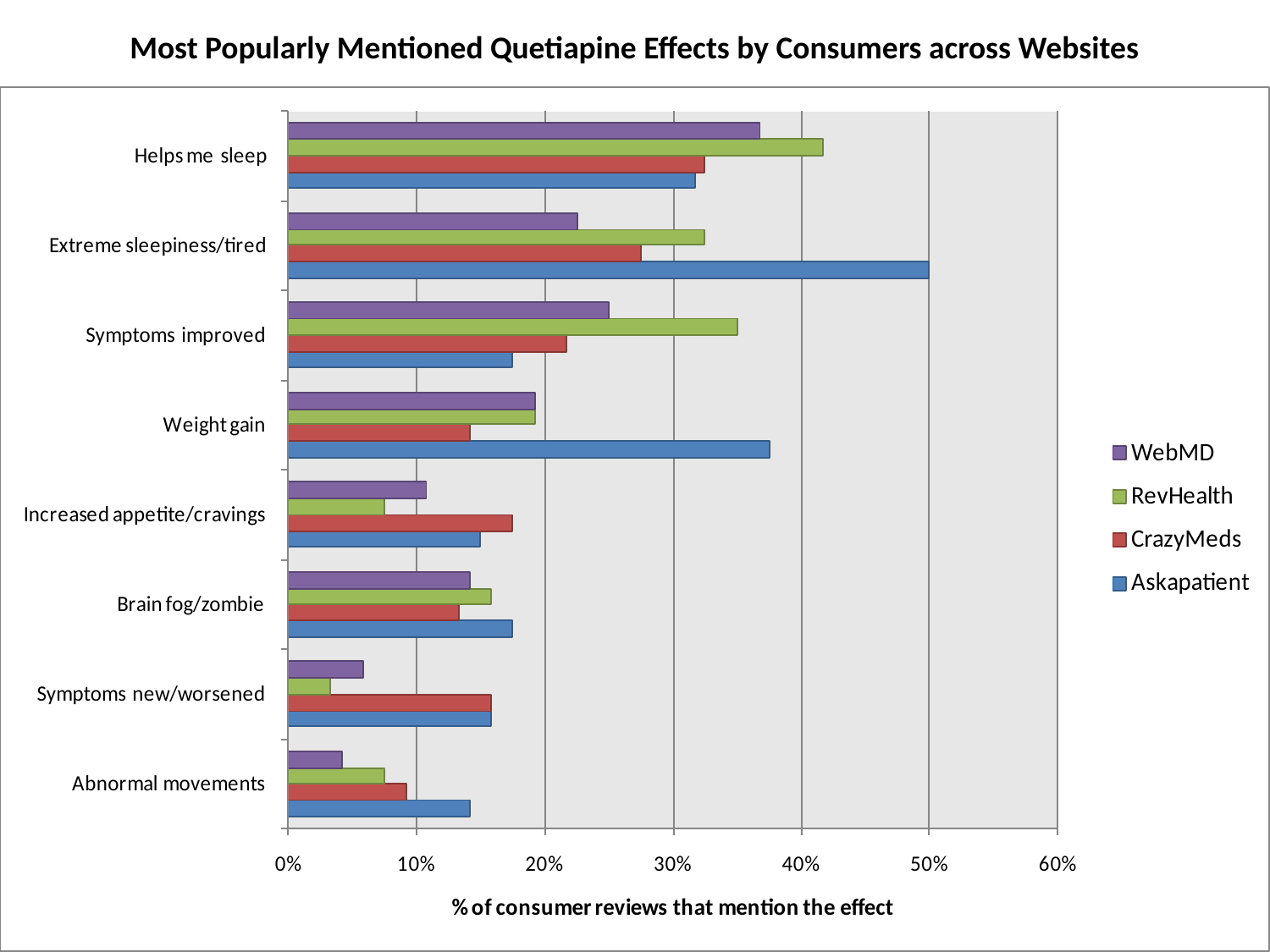

Most Popularly Mentioned Quetiapine Effects by Consumers across Websites

## Slide 11
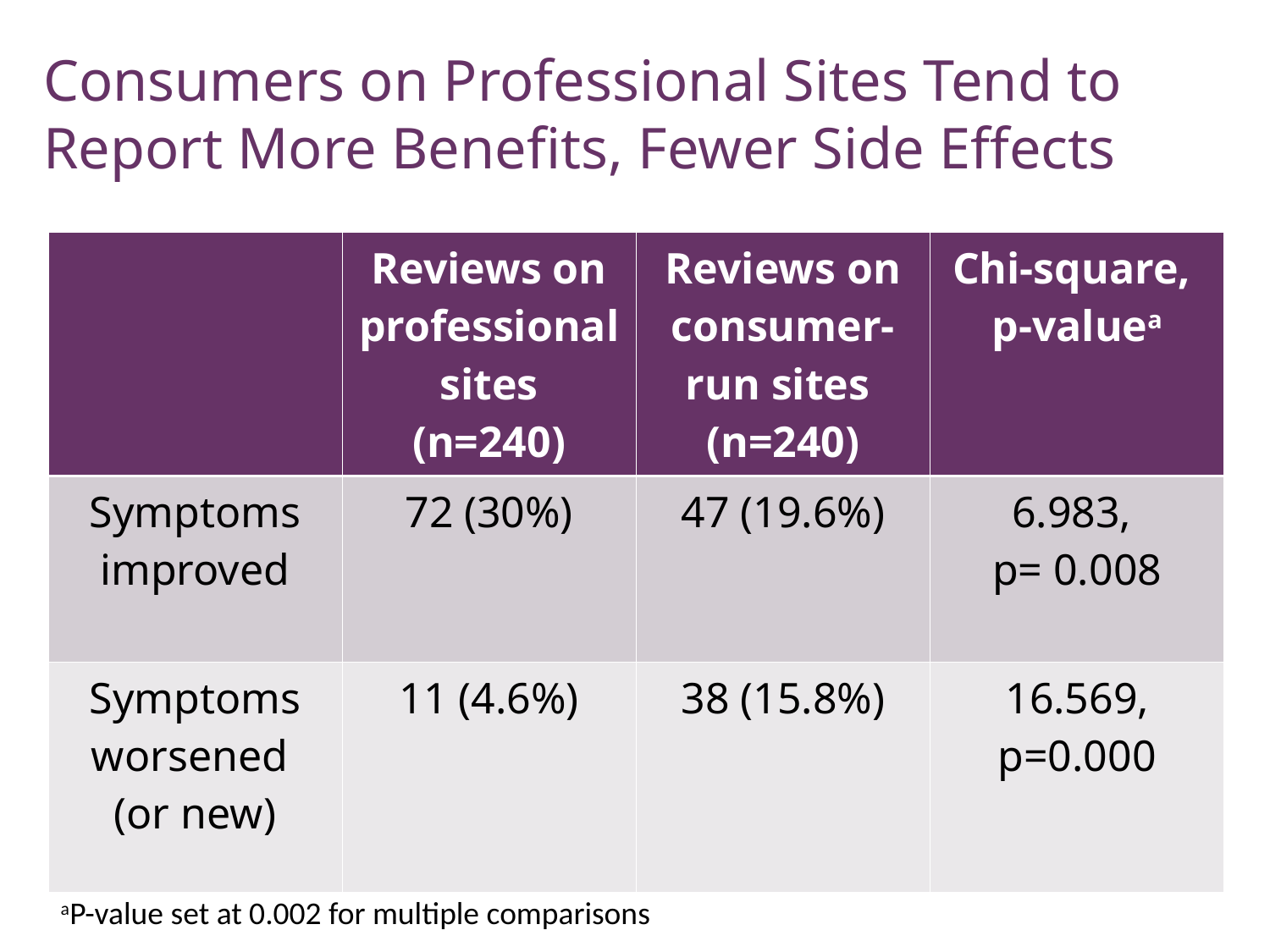

# Consumers on Professional Sites Tend to Report More Benefits, Fewer Side Effects
| | Reviews on professional sites (n=240) | Reviews on consumer-run sites (n=240) | Chi-square, p-valuea |
| --- | --- | --- | --- |
| Symptoms improved | 72 (30%) | 47 (19.6%) | 6.983, p= 0.008 |
| Symptoms worsened (or new) | 11 (4.6%) | 38 (15.8%) | 16.569, p=0.000 |
aP-value set at 0.002 for multiple comparisons

## Slide 12
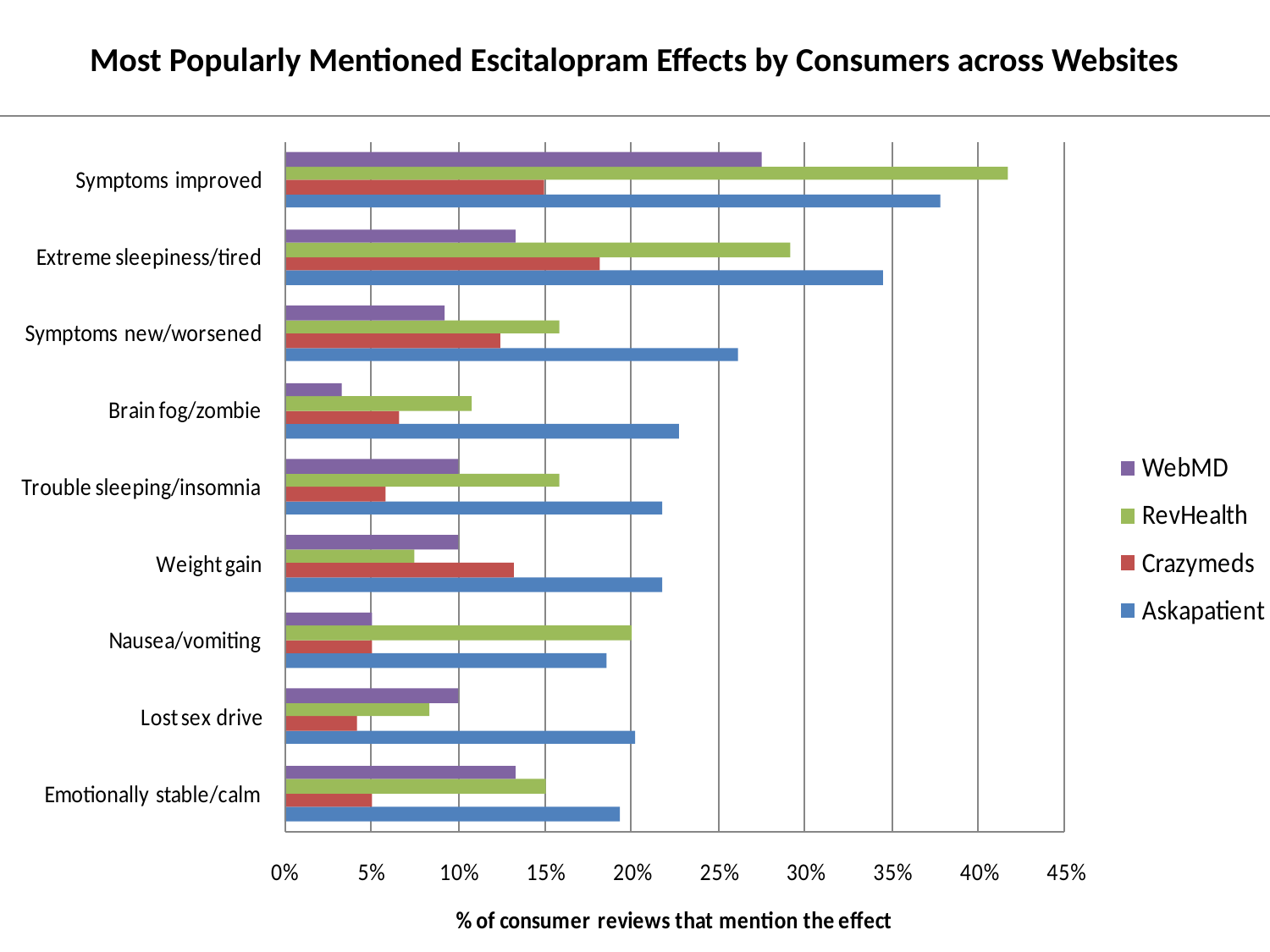

Most Popularly Mentioned Escitalopram Effects by Consumers across Websites

## Slide 13
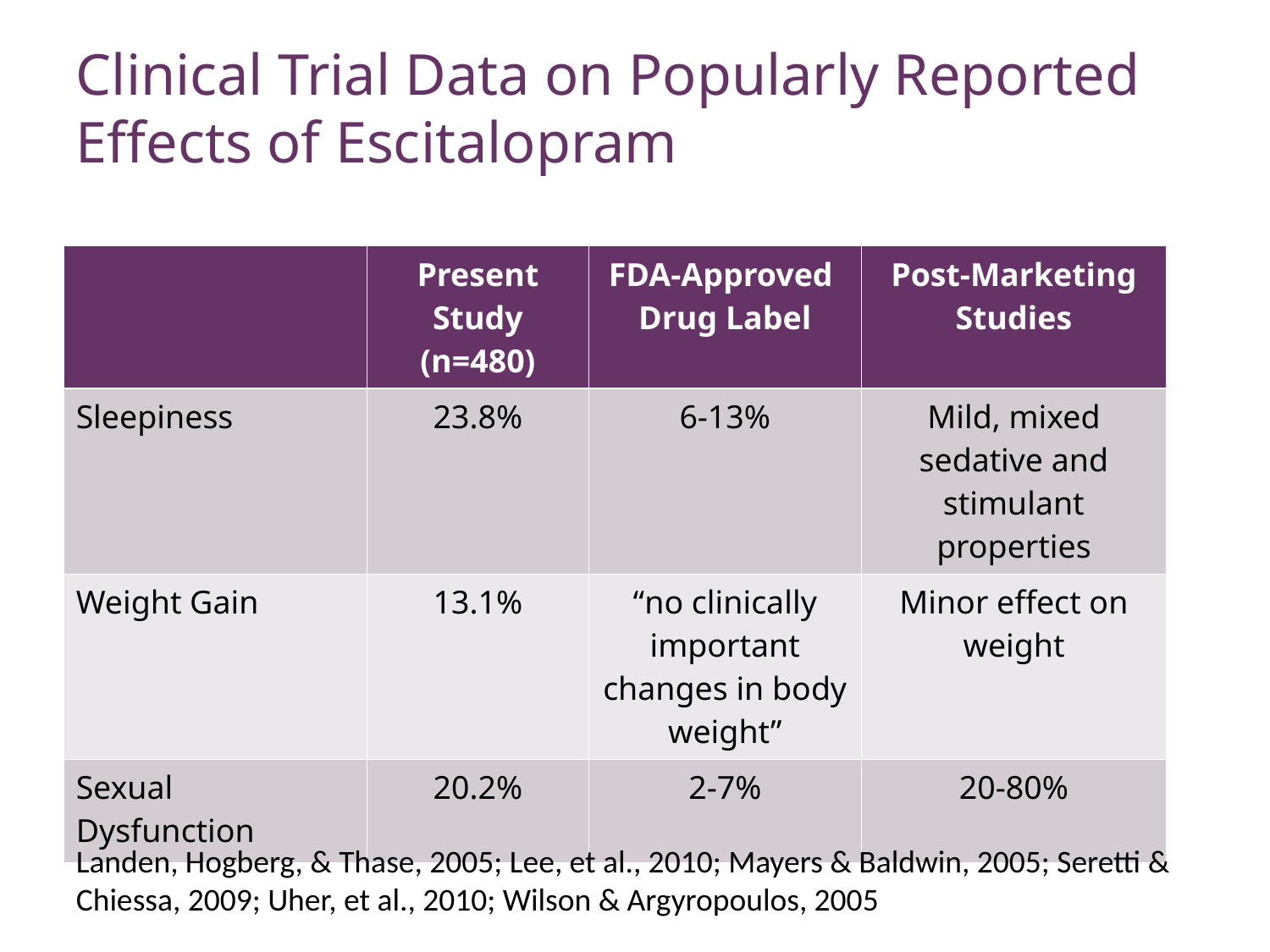

# Clinical Trial Data on Popularly Reported Effects of Escitalopram
| | Present Study (n=480) | FDA-Approved Drug Label | Post-Marketing Studies |
| --- | --- | --- | --- |
| Sleepiness | 23.8% | 6-13% | Mild, mixed sedative and stimulant properties |
| Weight Gain | 13.1% | “no clinically important changes in body weight” | Minor effect on weight |
| Sexual Dysfunction | 20.2% | 2-7% | 20-80% |
Landen, Hogberg, & Thase, 2005; Lee, et al., 2010; Mayers & Baldwin, 2005; Seretti & Chiessa, 2009; Uher, et al., 2010; Wilson & Argyropoulos, 2005

## Slide 14
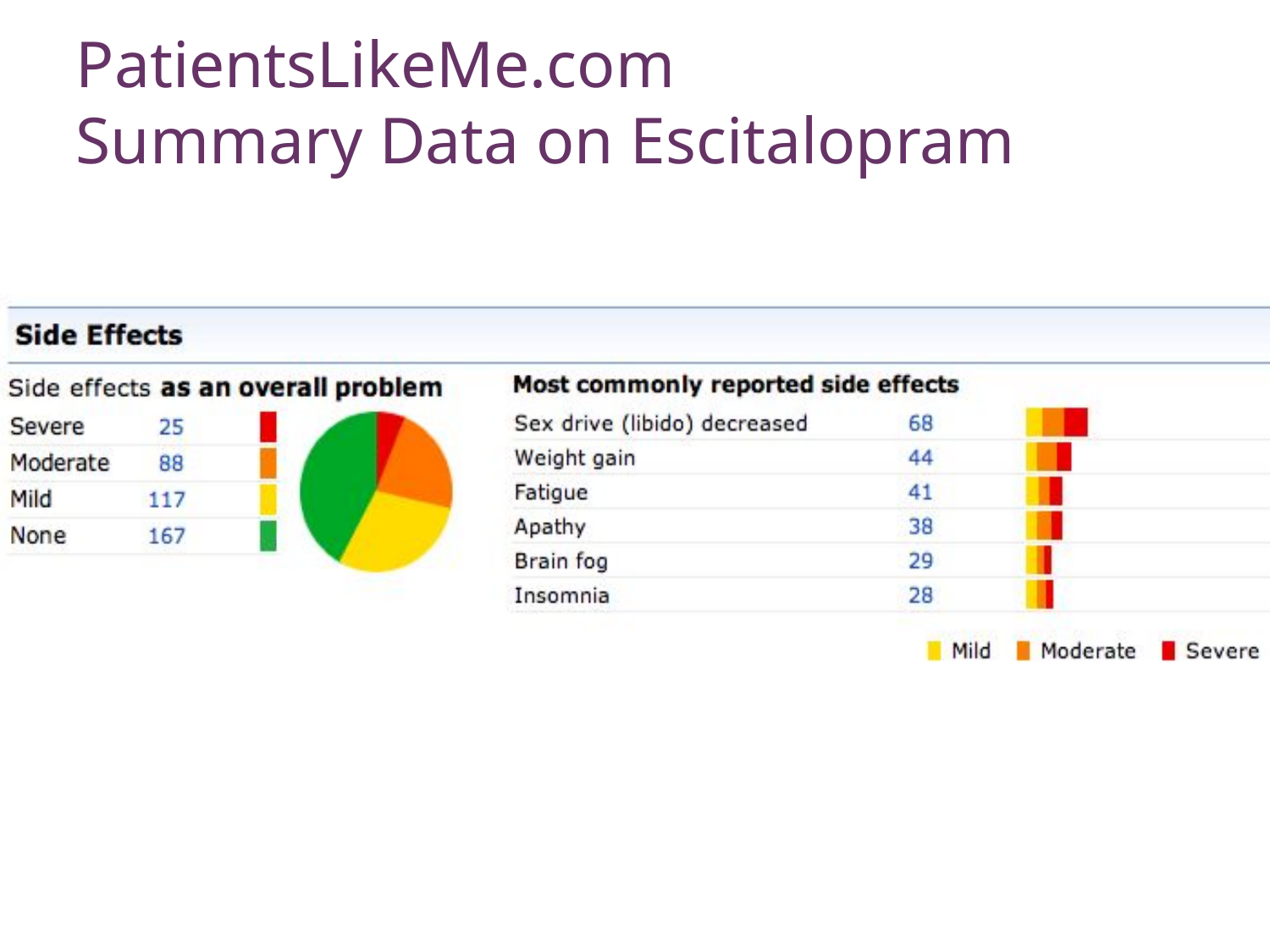

# PatientsLikeMe.com Summary Data on Escitalopram

## Slide 15
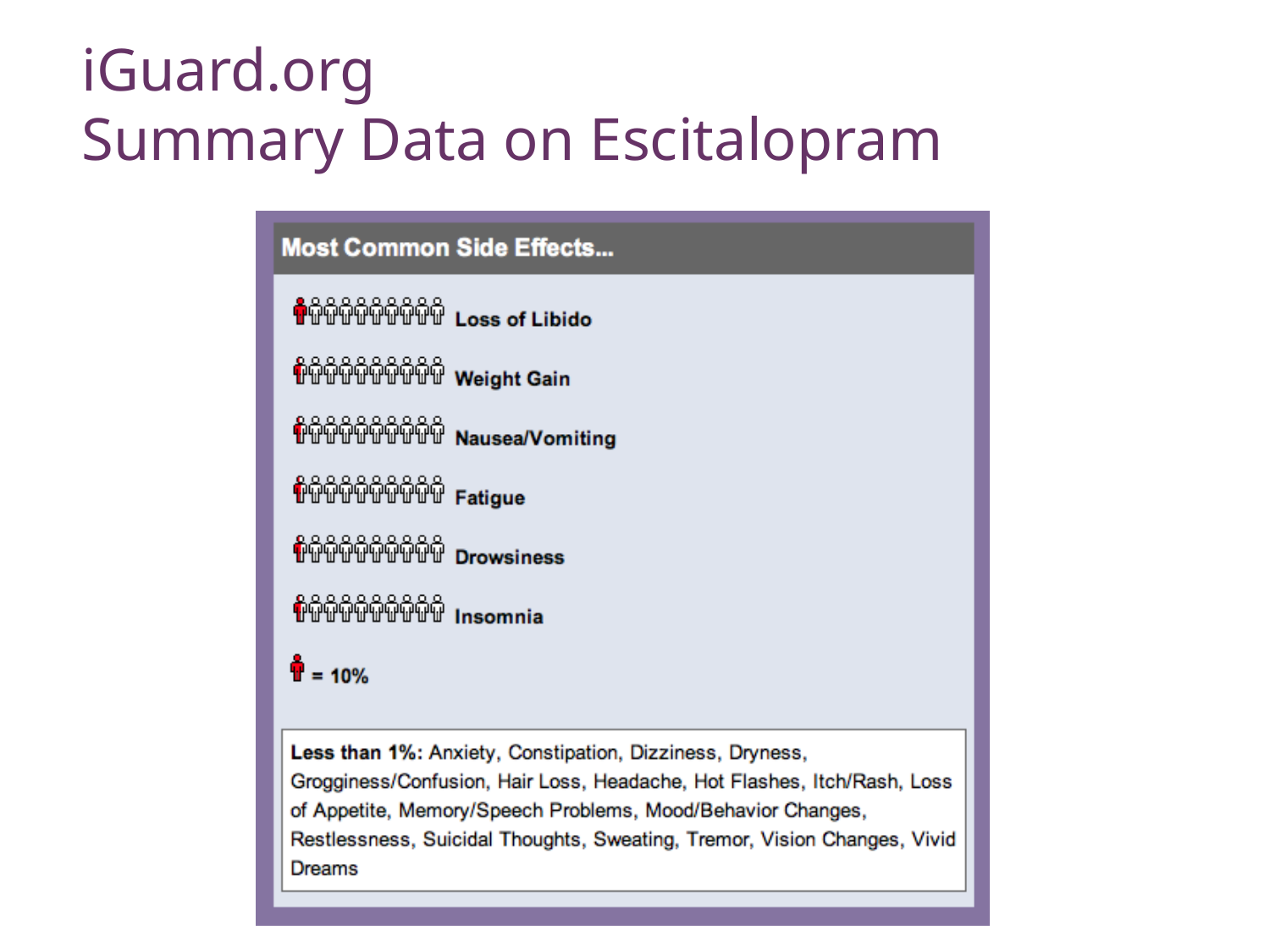

# iGuard.org Summary Data on Escitalopram

## Slide 16
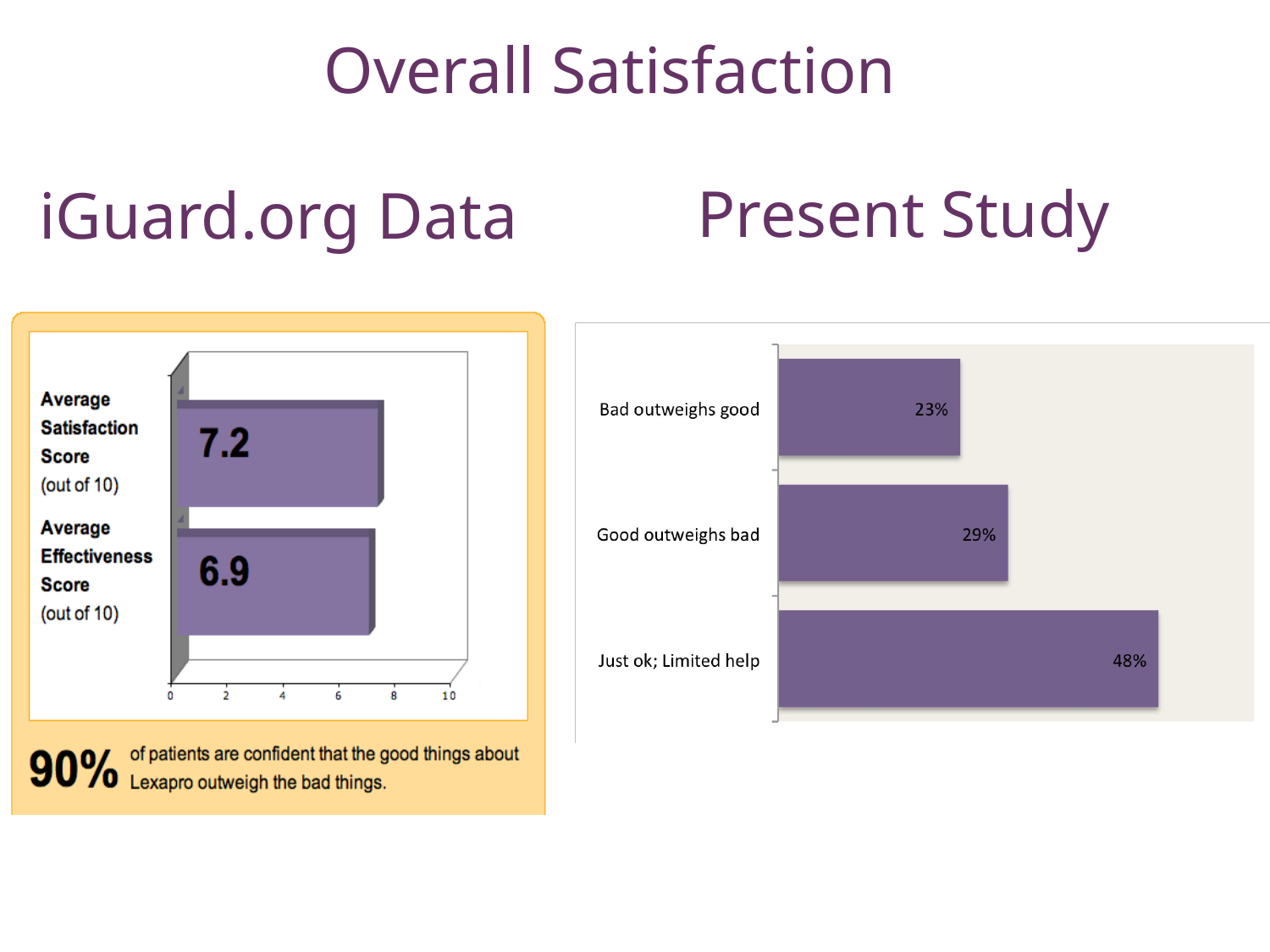

Overall Satisfaction
Present Study
# iGuard.org Data

## Slide 17
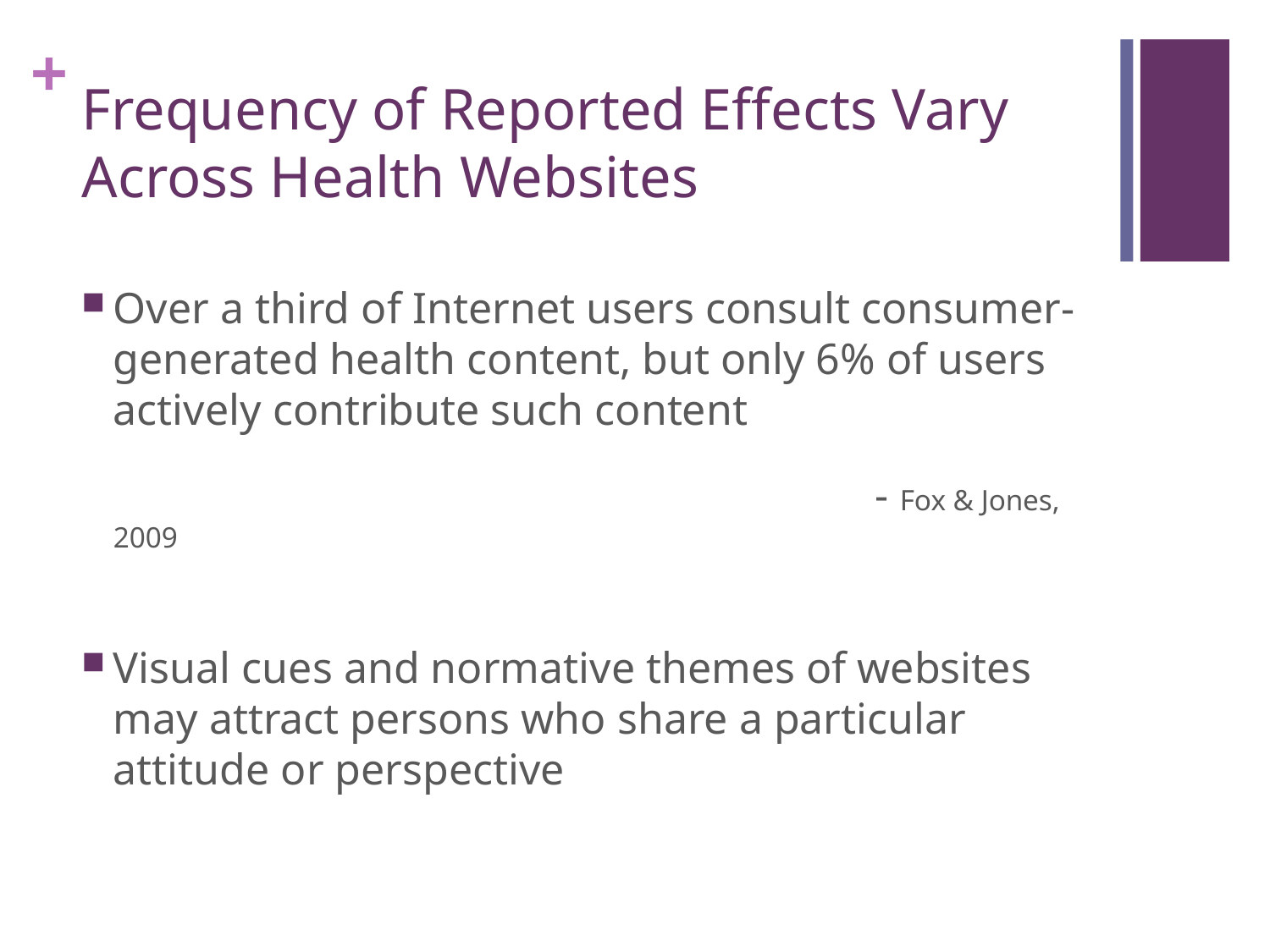

# Frequency of Reported Effects Vary Across Health Websites
Over a third of Internet users consult consumer-generated health content, but only 6% of users actively contribute such content
							- Fox & Jones, 2009
Visual cues and normative themes of websites may attract persons who share a particular attitude or perspective

## Slide 18
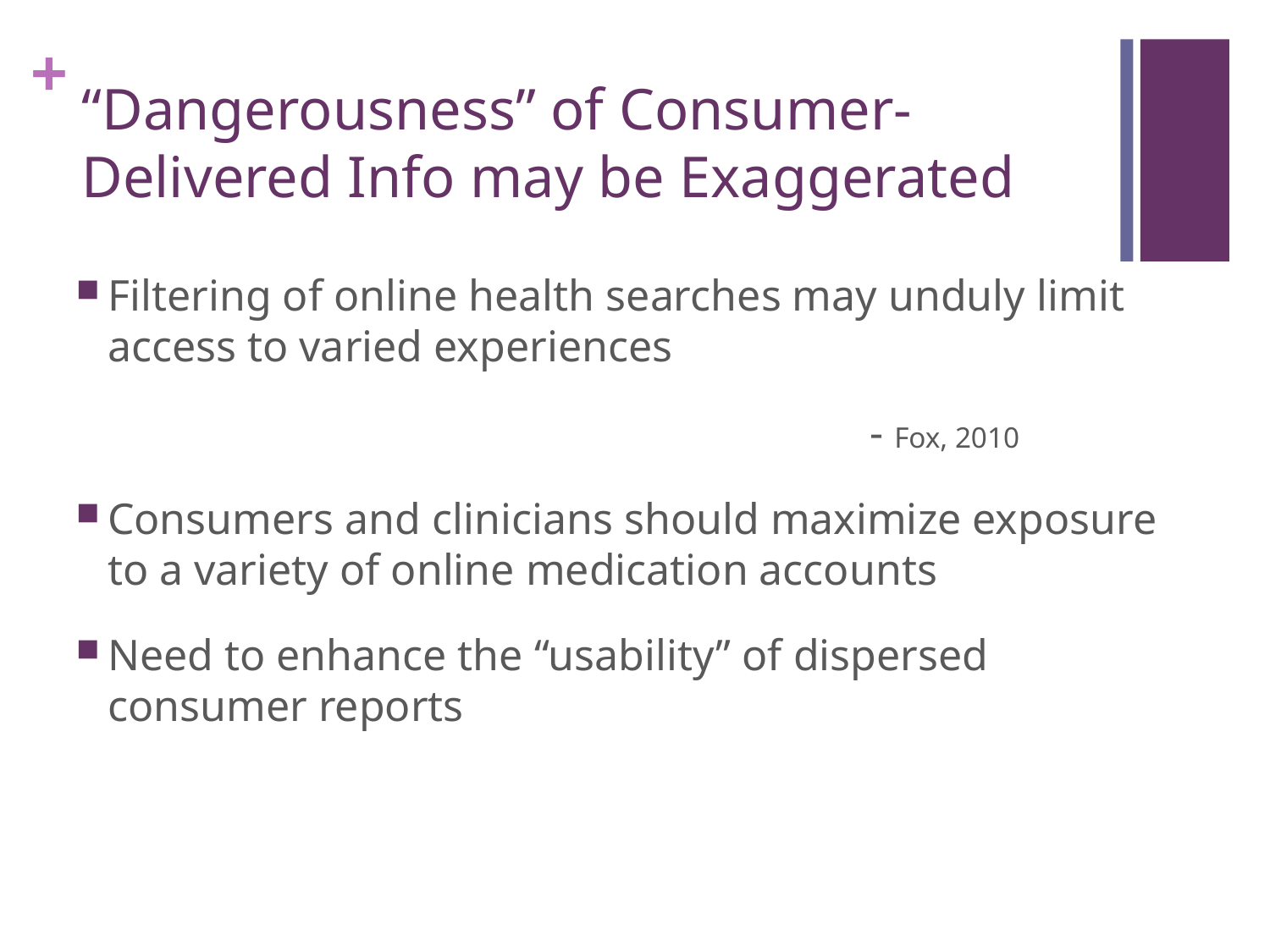

# “Dangerousness” of Consumer-Delivered Info may be Exaggerated
Filtering of online health searches may unduly limit access to varied experiences
							- Fox, 2010
Consumers and clinicians should maximize exposure to a variety of online medication accounts
Need to enhance the “usability” of dispersed consumer reports

## Slide 19
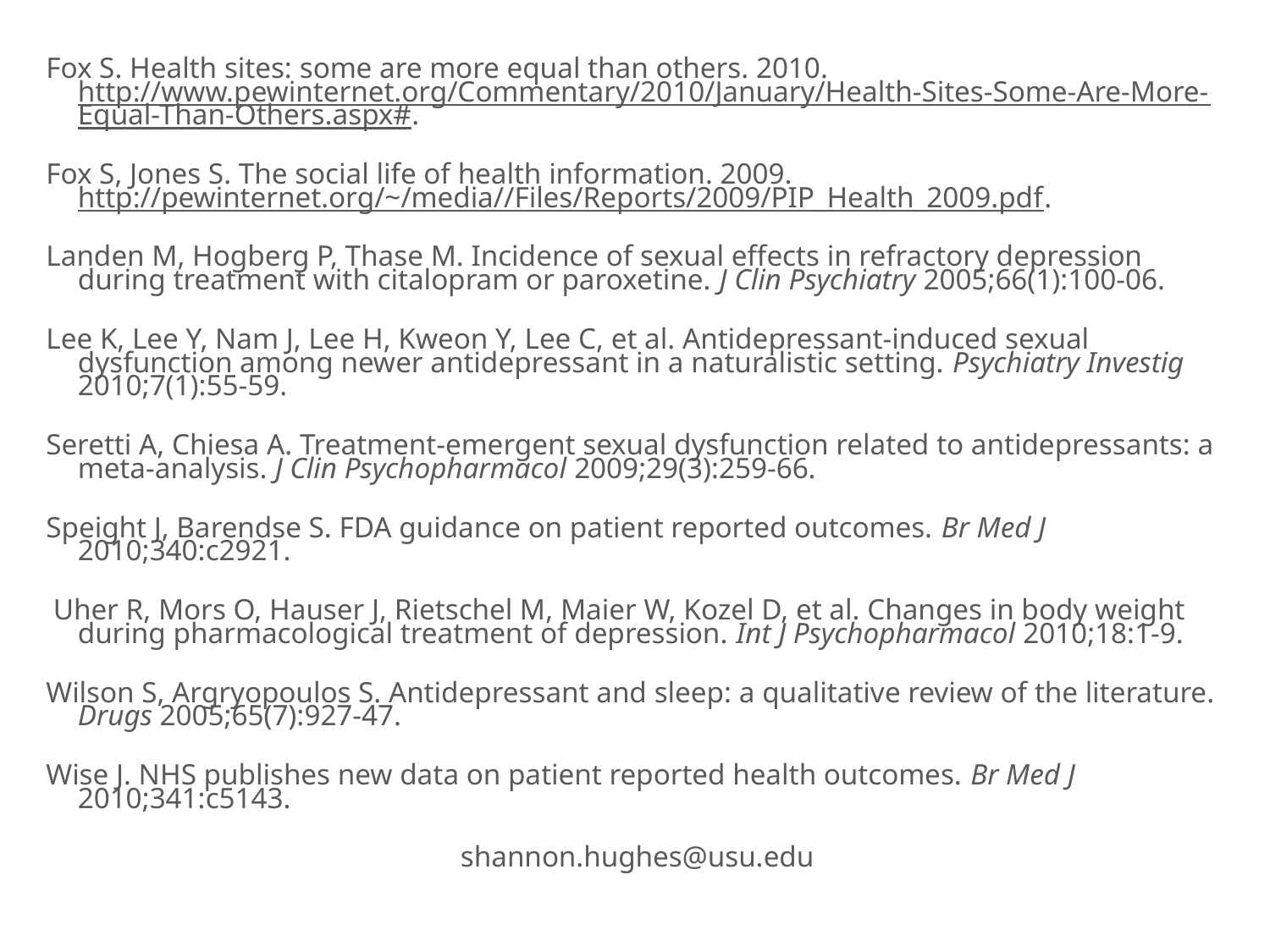

Fox S. Health sites: some are more equal than others. 2010. http://www.pewinternet.org/Commentary/2010/January/Health-Sites-Some-Are-More-Equal-Than-Others.aspx#.
Fox S, Jones S. The social life of health information. 2009. http://pewinternet.org/~/media//Files/Reports/2009/PIP_Health_2009.pdf.
Landen M, Hogberg P, Thase M. Incidence of sexual effects in refractory depression during treatment with citalopram or paroxetine. J Clin Psychiatry 2005;66(1):100-06.
Lee K, Lee Y, Nam J, Lee H, Kweon Y, Lee C, et al. Antidepressant-induced sexual dysfunction among newer antidepressant in a naturalistic setting. Psychiatry Investig 2010;7(1):55-59.
Seretti A, Chiesa A. Treatment-emergent sexual dysfunction related to antidepressants: a meta-analysis. J Clin Psychopharmacol 2009;29(3):259-66.
Speight J, Barendse S. FDA guidance on patient reported outcomes. Br Med J 2010;340:c2921.
 Uher R, Mors O, Hauser J, Rietschel M, Maier W, Kozel D, et al. Changes in body weight during pharmacological treatment of depression. Int J Psychopharmacol 2010;18:1-9.
Wilson S, Argryopoulos S. Antidepressant and sleep: a qualitative review of the literature. Drugs 2005;65(7):927-47.
Wise J. NHS publishes new data on patient reported health outcomes. Br Med J 2010;341:c5143.
shannon.hughes@usu.edu
